# Identification of a p53 target, *CD137L*, that mediates growth suppression and immune response of osteosarcoma cells

**DOI:** 10.1038/s41598-017-11208-x

**Published:** 2017-09-06

**Authors:** Yusuke Tsuda, Chizu Tanikawa, Takafumi Miyamoto, Makoto Hirata, Varalee Yodsurang, Yao-zhong Zhang, Seiya Imoto, Rui Yamaguchi, Satoru Miyano, Hiroshi Takayanagi, Hirotaka Kawano, Hidewaki Nakagawa, Sakae Tanaka, Koichi Matsuda

**Affiliations:** 1 Laboratory of Clinical Genome Sequencing, Department of Computational Biology and Medical Sciences, Graduate School of Frontier Sciences, University of Tokyo, Tokyo, Japan; 2 Department of Orthopedic Surgery, University of Tokyo, Tokyo, Japan; 3 Laboratory of Molecular Medicine, Human Genome Center, Institute of Medical Science, University of Tokyo, Tokyo, Japan; 4 Laboratory of DNA information Analysis, Human Genome Center, Institute of Medical Science, University of Tokyo, Tokyo, Japan; 5 Health Intelligence Center, Institute of Medical Science, University of Tokyo, Tokyo, Japan; 6 Department of Immunology, Graduate School of Medicine and Faculty of Medicine, University of Tokyo, Tokyo, Japan; 7 Department of Orthopedic Surgery, University of Teikyo, Tokyo, Japan; 8 Laboratory for Genome Sequencing Analysis, RIKEN Center for Integrative Medical Sciences, Tokyo, Japan

## Abstract

*p53* encodes a transcription factor that transactivates downstream target genes involved in tumour suppression. Although osteosarcoma frequently has *p53* mutations, the role of *p53* in osteosarcomagenesis is not fully understood. To explore p53-target genes comprehensively in calvarial bone and find out novel druggable p53 target genes for osteosarcoma, we performed RNA sequencing using the calvarial bone and 23 other tissues from *p53* ^+/+^ and *p53* ^−/−^ mice after radiation exposure. Of 23,813 genes, 69 genes were induced more than two-fold in irradiated *p53* ^+/+^ calvarial bone, and 127 genes were repressed. Pathway analysis of the p53-induced genes showed that genes associated with cytokine-cytokine receptor interactions were enriched. Three genes, *CD137L*, *CDC42 binding protein kinase gamma* and *Follistatin*, were identified as novel direct p53 target genes that exhibited growth-suppressive effects on osteosarcoma cell lines. Of the three genes, costimulatory molecule *Cd137l* was induced only in calvarial bone among the 24 tissues tested. CD137L-expressing cells exhibited growth-suppressive effects *in vivo*. In addition, recombinant Fc-fusion Cd137l protein activated the immune response *in vitro* and suppressed osteosarcoma cell growth *in vivo*. We clarified the role of CD137L in osteosarcomagenesis and its potential therapeutic application. Our transcriptome analysis also indicated the regulation of the immune response through p53.

## Introduction

Among the cancer-related genes that have been identified, *p53* is the most frequently mutated gene in human cancers^1^. *p53* encodes a transcription factor that transactivates downstream target genes involved in various cellular functions, including apoptosis^1^, cell cycle arrest^2^, metabolism^3^, stem cell maintenance^4^, and metastasis^5^, Through the regulation of these cellular functions, p53 plays a pivotal role in tumour suppression. In addition to these functions, emerging evidence has illuminated a new role of p53 in the regulation of the immune system^6^.

Osteosarcoma is the most common primary malignant bone tumour^7^. Comprehensive genome analyses of osteosarcoma have revealed that the most frequent mutation is that of the *p53* gene (up to 80% of cases)^8–12^. The association between p53 inactivation and osteosarcomagenesis is also observed in patients with Li-Fraumeni syndrome, an autosomal dominant disorder characterized by a germline mutation in *p53*, who have a higher risk of developing osteosarcoma^13^. Moreover, deletion of *p53* in mouse osteoblast has been reported to result in the development of osteosarcoma, and osteoblast or osteoblast precursor in bone is considered to be cells of origin in osteosarcoma^7, 14^. Thus, p53 behaves as a core tumour suppressor in osteosarcoma. However, the roles of *p53* in the pathogenesis of osteosarcoma are not fully understood.

Recent genome-wide profiling of p53 binding and transcriptional activity has shown that the precise cellular responses triggered by p53 are cell-type dependent^15^. Moreover, various malignant tumours occur in patients with Li-Fraumeni syndrome or *p53* ^−/−^ mice, and the incidence of these tumours differs^13, 16^. These results suggest that the tumour suppressive roles of *p53* are organ- or cell-type dependent. In these contexts, unravelling the comprehensive p53 functions specific to bone or osteoblasts is crucial to elucidate the roles of *p53* in osteosarcomagenesis.

Current therapies for osteosarcoma incorporate surgical resection and combination chemotherapy (doxorubicin, cisplatin and methotrexate), which cures approximately 70% of patients^17^. However, survival for patients with metastatic or relapsed osteosarcoma has remained virtually unchanged over the past 30 years, with an overall 5-year survival rate of approximately 20%^18^. Consequently, new therapies are needed. Because the restoration of wild-type p53 function in osteosarcoma cells has not succeeded clinically owing to the complexity of p53 signalling^1^, identification of druggable p53 downstream molecules or pathways could be key to attacking *p53*-deficient tumours such as osteosarcoma.

Therefore, we aimed to clarify p53 target genes comprehensively in calvarial bone which mainly consists of osteoblasts using RNA sequencing and to find novel p53 target genes, especially for bone-specific p53 targets. In addition, the purpose of this study was to reveal the roles of p53 in osteosarcomagenesis and to identify novel druggable p53 targets.

## Results

### p53 target genes in calvarial bone and enriched pathways

**Figure 1:**
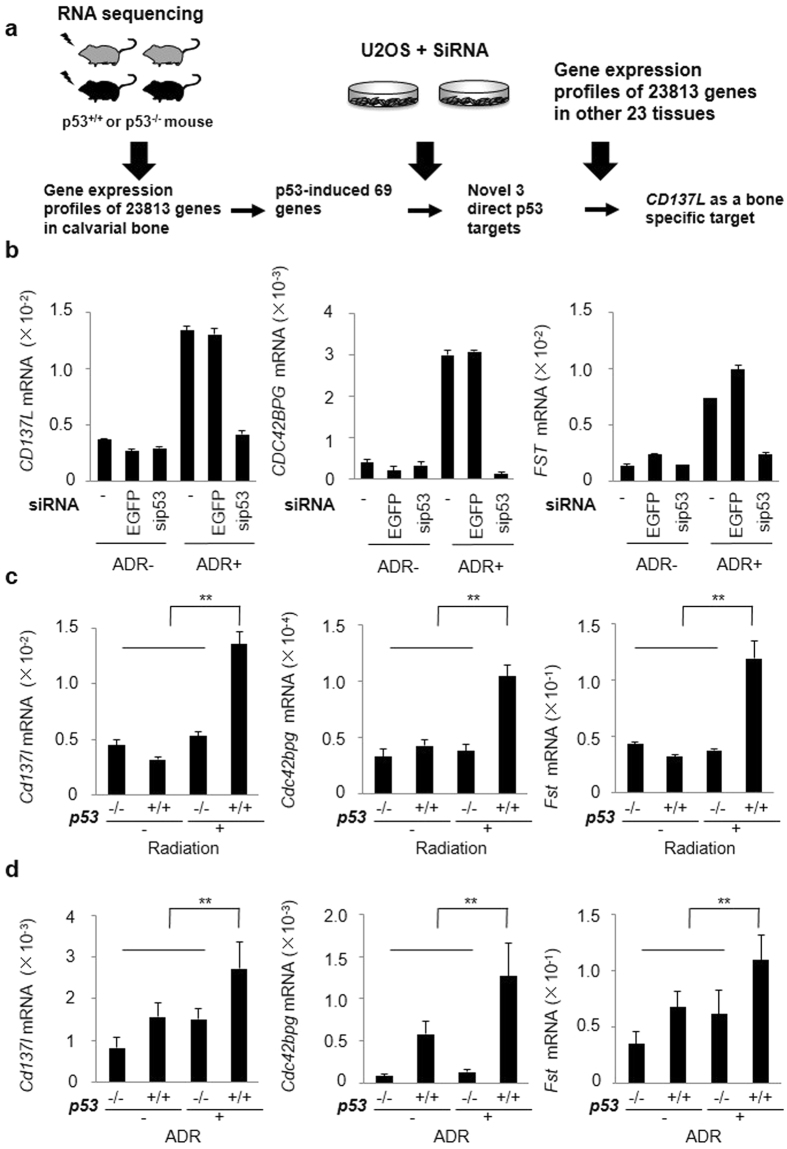
Regulation of *CD137L*, *CDC42BPG* and *FST* by p53. (**a**) Outline of the screening process. The expression profiles of 23813 genes in calvarial bone were detected by RNA sequencing, and 69 genes were selected by the indicated criteria as p53-induced genes. A second screening revealed three novel direct p53 targets. Of the 3 genes, one gene (*CD137L*) was up-regulated specifically in bone among 24 tissues. (**b**) At 24 h after transfection of each siRNA, U2OS cells were treated with ADR (2 μg/ml for 2 h). At 36 h after treatment, qPCR was performed. siRNA against *EGFP* was used as a control. β-actin was used for the normalization of expression levels. Error bars represent SD (n = 2). (**c**) qPCR was performed for the same calvaria sample as the RNA sequencing. β-actin was used for normalization of the expression levels. Error bars represent SD (n = 3). **P < 0.001, Student’s t-test. (**d**) qPCR was performed 36 h after treatment with ADR (2 μg/ml for 2 h) in *p53* ^+/+^ or *p53* ^−/−^ calvarial osteoblasts. β-actin was used for normalization of the expression levels. Error bars represent SD (n = 3). **P < 0.001, Student’s t-test.

We conducted RNA sequencing of 280 samples from 24 tissues including calvarial bone of *p53* ^+/+^ and *p53* ^−/−^ mice^19^. *p53* ^+/+^ and *p53* ^−/−^ mice were sacrificed for organ sampling 24 h after 10 Gy of whole-body X-ray irradiation. Samples were categorized into 4 groups: (K) *p53* ^−/−^ mice without irradiation, (W) *p53* ^+/+^ mice without irradiation, (KX) *p53* ^−/−^ mice with irradiation, and (WX) *p53* ^+/+^ mice with irradiation (n = 3 per group). First, we analyzed mRNA expression profiles of 23,813 transcripts in 12 calvarial bone samples (K, W, KX, WX, n = 3 per group) (Fig. 1a).

We screened genes whose expression level was induced or repressed only in the WX group. Of 23813 genes, 69 genes were induced more than two-fold in *p53* ^+/+^ mice after radiation exposure compared with the other groups (Supplementary Table 1a). Representative p53 target genes including *p21*, *Bax* and *Fas* were induced by radiation in *p53* ^+/+^ mice (Supplementary Fig. 1a). Pathway analysis for the 69 induced genes showed that genes related to the p53 signalling pathway, cytokine-cytokine receptor interactions, the TGF-β signalling pathway, and apoptosis were enriched (Table 1). On the other hand, the expression of 127 genes was more than two-fold repressed (Supplementary Table 1b). Enriched pathway of p53-repressed genes included the cell cycle pathway. p53-binding peak was detected in 49 genes (71%) among 69 induced genes in CHIP sequence data^20^, while p53-binding peak was detected in only 8 genes (6%) among 127 repressed genes. Moreover, 51 of 127 genes were shown to be regulated by p53-p21-DREAM pathway in the previous report^21^. Therefore, many of down-regulated genes are likely to be regulated through indirect mechanisms.

**Table 1:** Pathway analysis for the p53-induced and p53-repressed genes.

	Term	Genes	**−**log (p value)	Fold enrichment
p53-induced genes	p53 signalling pathway	*P21*, *Bbc3*, *Z3*, *Bax*, *Rprm*, *Mdm2*, *Pmaip1*, *Fas*, *Perp*, *Sesn2*, *Ccng1*	12.27	30.49
	Cytokine-cytokine receptor interaction	*Inhbb*, *Tnfrsf10b*, *Gdf5*, *Eda2r*, *Fas*, *Cd137l*	2.16	4.7
	TGF-beta signalling pathway	*Inhbb*, *Gdf5*, *Fst*	1.15	6.6
	Apoptosis	*Tnfrsf10b*, *Bax*, *Fas*	1.15	6.6
p53-repressed genes	Cell cycle	*Cdk1*, *E2f2*, *Cdc6*, *Ttk*, *Chek1*, *Mcm2*, *Mcm3*, *Mcm5*, *Mcm6*, *Ccne2*, *Cdc45*, *Ccnb2*, *Plk1*, *Bub1*, *Bub1b*, *Ccna2*	10.99	21.1
	DNA replication	*Prim1*, *Dna2*, *Pole*, *Mcm2*, *Mcm3*, *Mcm5*, *Mcm6*	5.70	33.75
	Oocyte meiosis	*Ccne2*, *Cdk1*, *Ccnb2*, *Plk1*, *SgoL1*, *Bub1*, *Fbxo5*	2.82	10.27
	p53 signalling pathway	*Ccne2*, *Cdk1*, *Ccnb2*, *Rrm2*, *Chek1*	2.44	12.23
	Progesterone-mediated oocyte maturation	*Cdk1*, *Ccnb2*, *Plk1*, *Bub1*, *Ccna2*	1.81	9.93
	Pyrimidine metabolism	*Prim1*, *Rrm2*, *Pole*, *Tk1*	1.54	7.03

### Screening of novel p53-target genes in calvarial bone by RNA sequencing

Of the 69 p53-induced genes, 28 have already been reported as direct p53 targets. With the exception of 10 genes that did not have a human homolog, we identified 31 candidate p53 novel target genes (Supplementary Table 1a). For the second screening to identify novel p53 direct target genes from the 31 candidate genes, we explored genes whose expression levels were induced by adriamycin (ADR) and changed in a p53-dependent manner using a human osteosarcoma cell line (Fig. 1a). At 24 h after transfection with small interfering RNA (siRNA) targeting p53 (sip53), U2OS osteosarcoma cells were treated with 2 µg/ml ADR for 2 h. Expression of *p21* (positive control) was induced by ADR treatment but supressed in sip53-treated cells compared with that of control cells (Supplementary Fig. 1b). We assessed the expression of all 31 candidates by quantitative real-time PCR (qPCR) and identified *CD137L*, *CDC42 binding protein kinase gamma* (*CDC42BPG*) and *Follistatin* (*FST*) as novel p53-regulated genes (Fig. 1b). We found that ADR induced the expression of the three genes in a dose-dependent manner in U2OS cells (Supplementary Fig. 1c). We also confirmed the expression of the three genes in mouse calvarial bone tissues by qPCR and observed their induction by X-ray irradiation only in the calvarial bone of *p53* ^+/+^ mice, concordant with our RNA sequencing analysis (Fig. 1c, Supplementary Fig. 1d).

Then, we evaluated the expression of these genes in calvaria-derived primary osteoblasts. We found that ADR treatment induced the mRNA expression of the *Cd137l*, *Cdc42bpg* and *Fst* genes in primary osteoblasts (Supplementary Fig. 1e). Moreover, *Cd137l*, *Cdc42bpg* and *Fst* mRNA levels were significantly increased in *p53* ^+/+^ primary osteoblasts with ADR than those of other groups (Fig. 1d). These findings clearly indicated that p53 regulates the expression of *Cd137l*, *Cdc42bpg* and *Fst* in response to DNA damage.

### Identification of CD137L as a novel bone-specific p53 target gene

**Figure 2:**
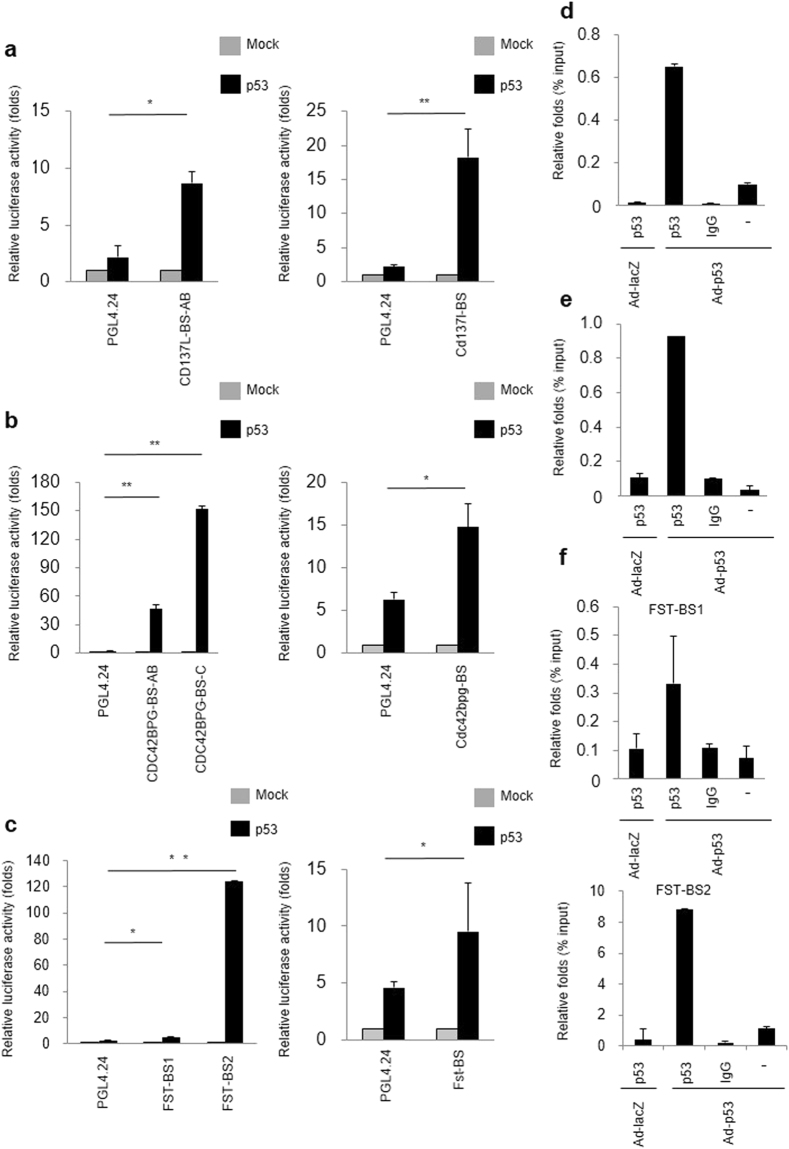
Identification of *CD137L*, *CDC42BPG* and *FST* as p53 direct target genes. (**a**–**c**) Luciferase assay of the p53BS in human (left) or mouse (right) *CD137L* (**a**), *CDC42BPG* (**b**) or *FST* (**c**) using SaOS2 cells. Luciferase activity is indicated relative to the activity of the mock vector with SD (n = 3). *P < 0.05, *P < 0.001, Student’s t-test. (**d**–**f**) CHIP assay for *CD137L* (**d**), *CDC42BPG* (BS-C) (**e**) or *FST* (**f**) was performed using SaOS2 cells that were infected with Ad-LacZ (lane 1) or Ad-p53 (lane 2-4). DNA-protein complexes were immunoprecipitated with an anti-p53 antibody (lanes 1 and 2) followed by qPCR. Input chromatin represents a small portion (1%) of the sonicated chromatin collected prior to immunoprecipitation. Immunoprecipitates with an anti-IgG antibody (lane 3) or in the absence of an antibody (lane 4) were used as negative controls. Columns, mean; error bars, SD (n = 3).

Subsequently, we surveyed the genomic sequences of the human and mouse *CD137L*, *CDC42BPG* and *FST* to detect p53-binding sequences (p53BS). The human and mouse *CD137L*, *CDC42BPG* and *FST* genes had potential p53BS within the first intron or promoter region (<5000 bp upstream from the transcription start site) (Supplementary Fig. 2a–c). We then subcloned a human or mouse *CD137L* DNA fragment, which included two putative p53BSs, into a pGL4.24 promoter vector (pGL4.24/CD137L-BS). We found that the co-transfection of pGL4.24/CD137L-BS with the wild-type p53 expression plasmid enhanced the luciferase activity (Fig. 2a). For the other 2 genes, co-transfection of pGL4.24/CDC42BPG-BS or pGL4.24/FST-BS also enhanced the luciferase activity (Fig. 2b,c). To examine the possible binding of p53 to these DNA segments, we carried out a chromatin immunoprecipitation (ChIP) assay using SaOS2 cells (p53-null) that were infected with either Ad-p53 or Ad-LacZ. qPCR of immunoprecipitated DNA indicated that the p53 protein bound to the genomic fragment containing the p53BSs (Fig. 2d–f). We analyzed published CHIP sequence data^22^ and found p53-binding peaks at 3.5-kb 3′ flanking region of the FST gene (FST-BS2). We cloned potential p53 binding sequence of FST gene (FST-BS2) and performed luciferase assay. As a result, p53 induced luciferase activity through this sequence (Fig. 2c). ChIP assay using SaOS2 cells also indicated the binding of p53 to this genomic fragment (Fig. 2f). These findings implied that p53 directly regulated the expression of the three genes through multiple p53BSs.

**Figure 3:**
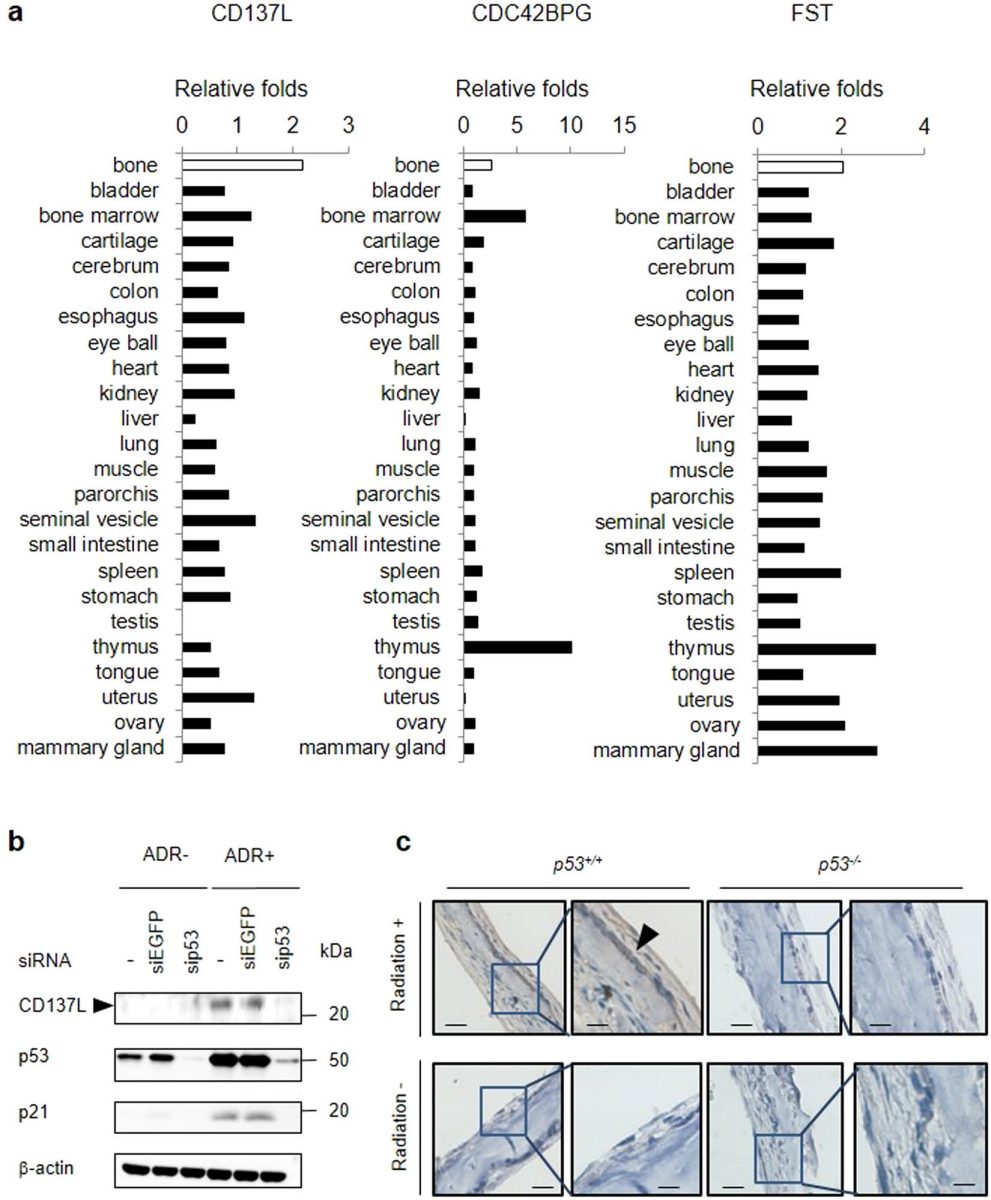
Identification of *CD137L* as a bone-specific p53 target gene. (**a**) Induction of *Cd137l* among 24 tissues. Samples were categorized into 4 groups: (K) *p53* ^−/−^ mice without irradiation, (W) *p53* ^+/+^ mice without irradiation, (KX) *p53* ^−/−^ mice with irradiation and (WX) *p53* ^+/+^ mice with irradiation (n = 3 per group). We calculated the median FPKM value of WX/maximum FPKM value of the median FPKM value in K, KX or W for 24 tissues. (**b**) At 24 h after transfection with each siRNA, U2OS cells were treated with ADR (2 μg/ml for 2 h). At 36 h after treatment, whole cell extracts were subjected to western blotting with an anti-CD137L, anti-p21, anti-p53, or anti-β-actin antibody. siRNA against EGFP was used as a control. β-actin was used for normalization of the expression levels. These images were cropped from full-length blots (Supplementary Fig. 9). (**c**) Immunohistochemical staining of Cd137l in mouse p53^α^ or p53^−/−^ calvaria with or without radiation exposure. Representative images from three tissues in each group are shown. Scale Bars (left) = 50 μm, Scale Bars (right) = 20 μm. Black arrowhead shows osteoblast.

To compare the expression of these genes in mouse 24 tissues, we calculated the median fragments per kilobase of exon per million mapped fragments (FPKM) value of WX/maximum FPKM value of the median K, median KX or median W for 24 tissues in our RNA sequencing data. We compared the fold change in the calvarial bone with those in the 23 other tissues. *Cd137l* was more than two-fold induced only in the calvarial bone among the 24 tissues analysed (Fig. 3a). *Cdc42bpg* and *Fst* did not show bone-specific induction. We also found that CD137L protein was induced by DNA damage in U2OS cells, and sip53 remarkably reduced the CD137L protein level (Fig. 3b). In addition, immunohistochemistry indicated the induction of Cd137l protein by X-ray irradiation in mouse calvarial osteoblasts of *p53* ^+/+^ mice (Fig. 3c). Thus, we selected CD137L for further functional analysis.

### Growth suppressive effect of p53 or CD137L

**Figure 4:**
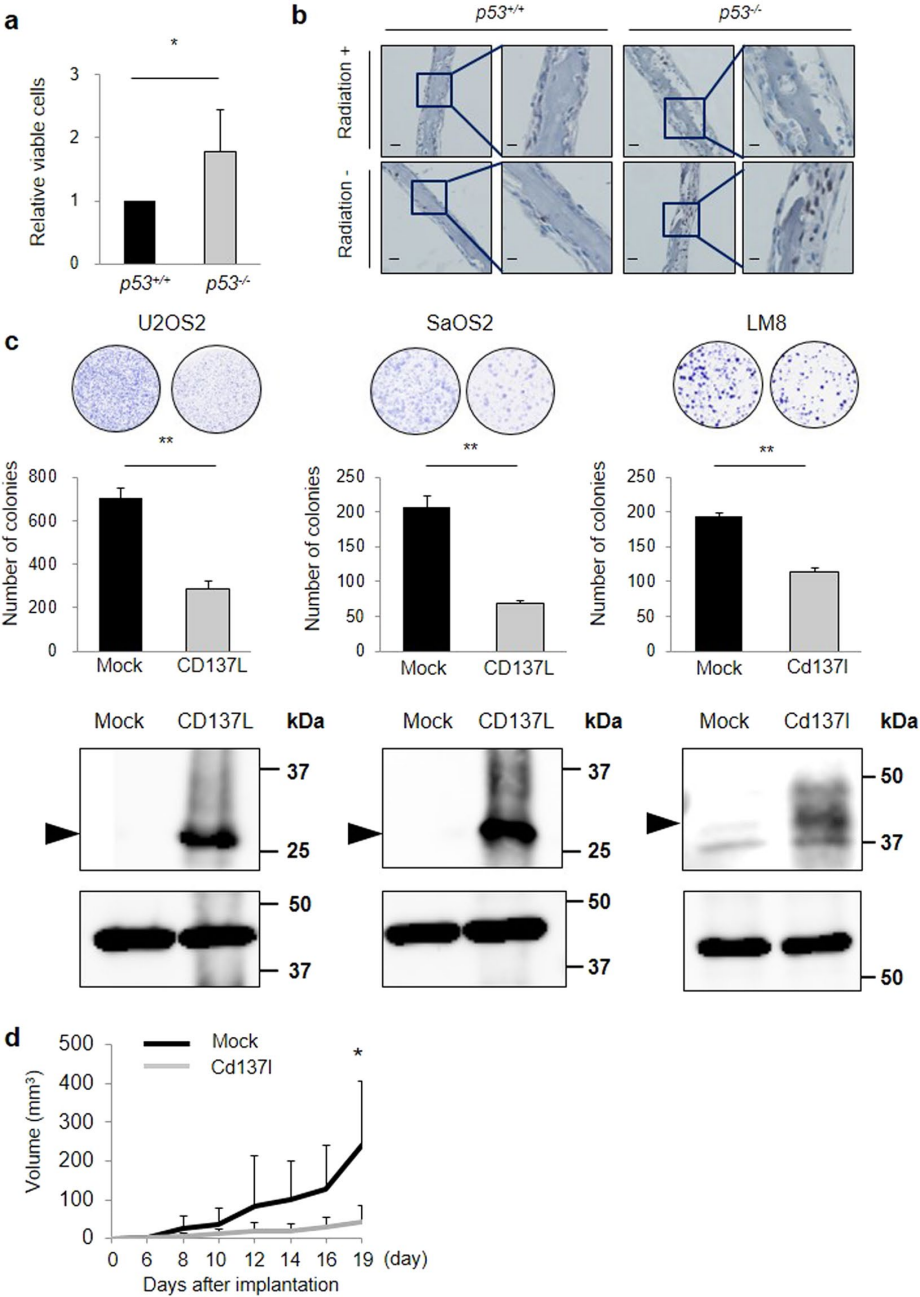
Growth suppressive effects of p53 or CD137L. (**a**) *p53* ^+/+^ calvarial osteoblasts or *p53* ^−/−^ calvarial osteoblasts were cultured on plates. Cell proliferation was assessed at 48 h. Error bars represent SD (n = 3). *P < 0.05, Student’s t-test. (**b**) Immunohistochemical staining of Ki67 in mouse p53^+/+^ or p53^−/−^ calvaria with or without radiation exposure. Representative images from three tissues in each group are shown. Scale Bars (left) = 50 μm, Scale Bars (right) = 20 μm. (**c**) A colony formation assay was performed. After ectopic expression of CD137L or mock, the number of SaOS2, U2OS or LM8 cells was determined. Whole cell extracts were subjected to western blotting with a human or mouse anti-CD137L antibody. Error bars represent SD (n = 3). **P < 0.001, Student’s t-test. β-actin (U2OS and SaOS2) and α-tubulin antibody (LM8) was used as loading control. (**d**) Cd137l (n = 3) or mock (n = 3) stable expression cell lines were inoculated into the left and right flanks of C3H mice; each group contains 2 mice. The tumour volume was calculated every 2 or 3 days. *P < 0.05, Wilcoxon rank-sum test.

Because osteoblast precursors are considered the cells of origin in osteosarcoma^7^, we evaluated the proliferation of primary osteoblasts. Primary osteoblasts from *p53* ^−/−^ mice showed increased proliferation compared with osteoblasts from *p53* ^+/+^ mice (Fig. 4a), as previously reported^23^. Ki67-positive osteoblasts were more frequently found in the *p53* ^−/−^ calvarial bone compared with those in *p53* ^+/+^ mice (Fig. 4b). In addition, Ki67-positive osteoblasts were not observed in the *p53* ^+/+^ calvarial bone with radiation exposure, demonstrating the growth suppressive effect of p53 in osteoblasts.

To analyse the function of *CD137L*, we carried out colony formation assays using human osteosarcoma cell lines (U2OS and SaOS2) and a mouse osteosarcoma cell line (LM8) transfected with plasmids expressing *CD137L* or mock. Ectopic expression of CD137L reduced cell proliferation, suggesting a growth-suppressive effect of CD137L (Fig. 4c). Moreover, ectopic expression of CDC42BPG, FST344 (major isoform of FST) or FST311 (minor isoform of FST) reduced cell proliferation, which indicated that these genes also had growth suppressive effects in osteosarcoma cell lines (Supplementary Fig. 3a,b).

Then, we investigated the growth suppressive effect of Cd137l *in vivo*. LM8 cells stably expressing mock or Cd137l (n = 3 each, Supplementary Fig. 4a,b) were subcutaneously inoculated into CH3 mice. As a result, Cd137l-expressing LM8 cells showed reduced tumour growth compared with mock-expressing LM8 cells (Fig. 4d, Supplementary Fig. 4c).

### Role of the CD137L reverse or direct signal in osteosarcoma

Although CD137L is a ligand of CD137, CD137L transmits a reverse signal through its interaction with CD137 and mediates diverse cellular responses, including proliferation and differentiation, in a variety of cells^24, 25^. We generated recombinant protein containing the extracellular domain of CD137 and the Fc region of human IgG protein (human CD137-Fc or mouse Cd137-Fc) or Cd137l and the Fc region of human IgG protein (mouse Cd137l-Fc) (Supplementary Fig. 5a,b). After transfection of HEK293 cells with plasmid expressing CD137L or mock, the cells were incubated with CD137-Fc or mock-Fc for 2 h. Interaction between CD137L and CD137-Fc was confirmed by immunocytochemistry using an anti-human IgG antibody (Supplementary Fig. 5c). We also confirmed the interaction between Cd137 and Cd137l-Fc.

**Figure 5:**
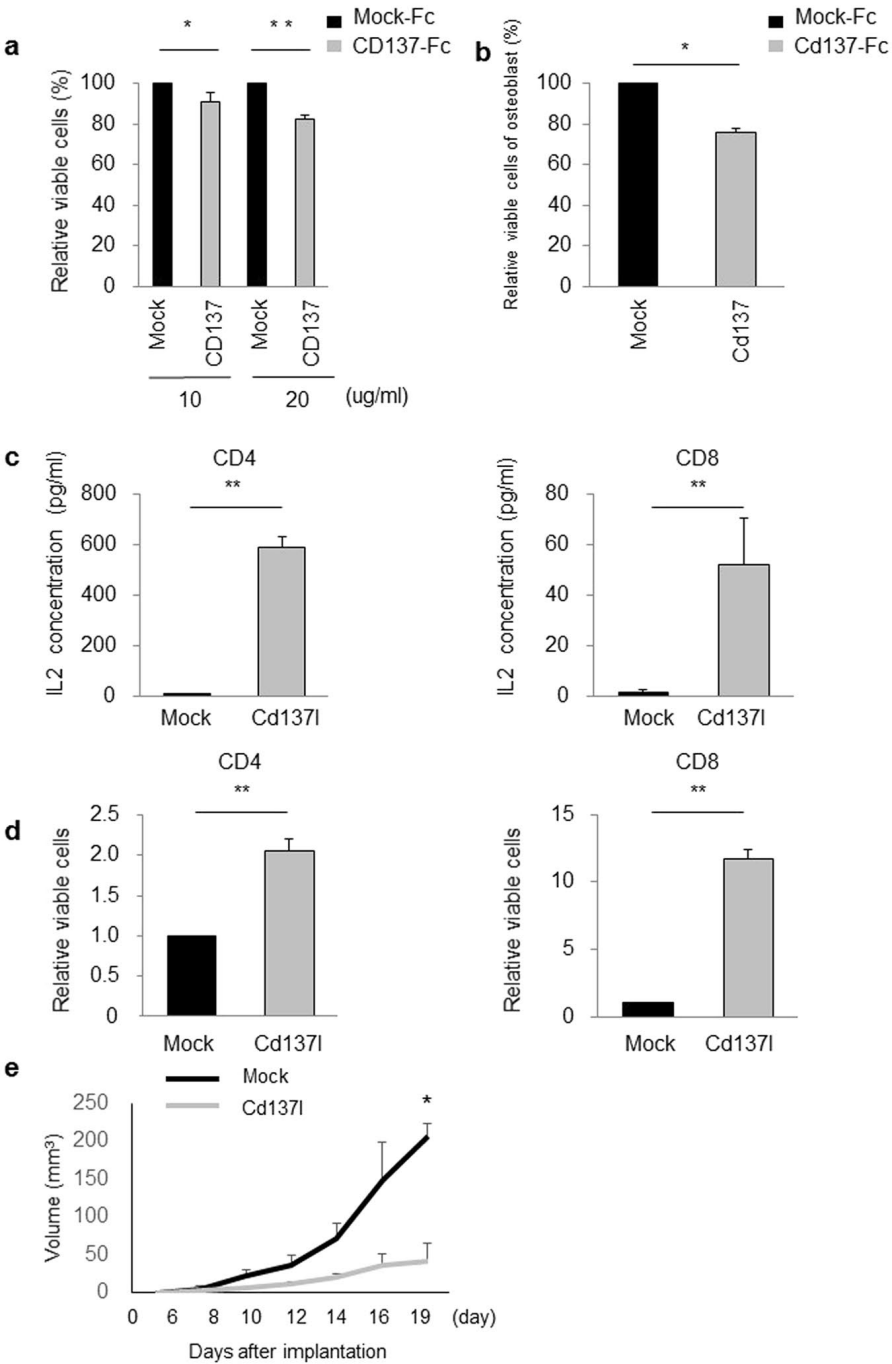
The role of CD137L reverse or direct signalling in osteosarcoma. (**a**) SaOS2 cells were cultured with 10 or 20 μg/ml CD137-Fc or mock-Fc. Cell proliferation was assessed at 48 h. Error bars represent SD (n = 3). *P < 0.05, **P < 0.001, Student’s t-test. (**b**) *p53* ^+/+^ calvarial osteoblasts were cultured with 40 μg/ml Cd137-Fc or mock-Fc protein. Cell proliferation was assessed at 48 h. Error bars represent SD (n = 3). *P < 0.05, Student’s t-test. (**c**,**d**) IL2 production (**c**) and proliferation (**d**) of CD4^+^ or CD8^+^ T cells were assessed 48 h after stimulation with Cd137l-Fc. **P < 0.001, Student’s t-test. (**e**) C3H/HeJ mice (each group, n = 3) were subcutaneously injected into the left flank with 0.1 ml of PBS containing 1 × 10^6^ LM8 cells. After tumours reached 0.5 cm in diameter, each of the mice was treated with Cd137l-Fc fusion protein or control mock-Fc by intraperitoneal injection for 3 days. The tumour volume was calculated every 2 or 3 days. Arrow shows the days of injection. *P < 0.05, Wilcoxon rank-sum test.

To investigate the effects of the CD137L reverse signalling, SaOS2 cells or osteoblasts were seeded on plates precoated with CD137-Fc or mock-Fc. CD137-Fc significantly reduced the proliferation of SaOS2 cells or *p53* ^+/+^ osteoblasts compared with cells treated with mock-Fc (Fig. 5a,b). These results indicate that CD137L reverse signalling inhibits the proliferation of osteosarcoma cells and osteoblasts.

The CD137L receptor/ligand system is used by antigen-presenting cells to costimulate T cell activity together with the T cell receptor/CD3 signal^26^. To investigate the effect CD137L on T cell activation, IL-2 production and cell proliferation were assessed using CD8^+^ and CD4^+^ T cells isolated from C3H/HeJ mice spleens. CD4^+^ and CD8^+^ cells were seeded in 24-well culture plates precoated with an anti-CD3 antibody in the presence of Cd137l-Fc or mock-Fc. Cd137l-Fc significantly induced IL-2 production and increased proliferation of CD8^+^ and CD4^+^ T cells (Fig. 5c,d).

Finally, we explored the effect of Cd137l-Fc on the growth of LM8 cells *in vivo*. Six-week-old male C3H/HeJ mice were subcutaneously injected with 1 × 10^6^ LM8 cells in the left flank. When tumours reached 0.5 cm in diameter, at approximately the 7th day after tumour implantation, mice (n = 3 each) were treated with 0.2 ml (50 µg) of Cd137l-Fc fusion proteins or control mock-Fc by intraperitoneal injection for 3 consecutive days. As a result, Cd137l-Fc injection significantly suppressed tumour growth at day19 compared with mock-Fc (Fig. 5e), demonstrating the growth suppressive effect of CD137/CD137L pathway.

## Discussion

**Figure 6:**
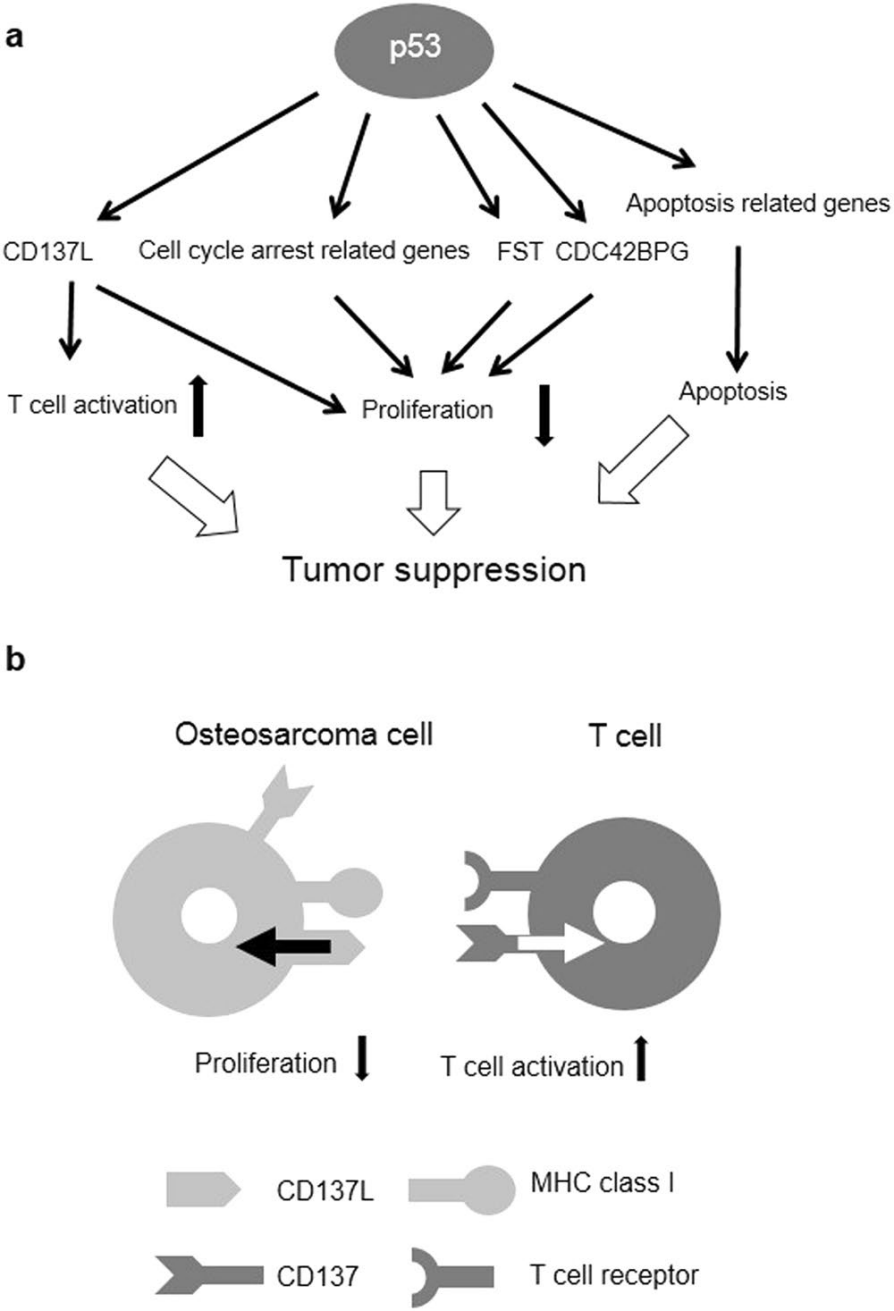
A schema of p53 or CD137L function. (**a**) A schema of the p53-regulated pathways or genes and the tumour suppressive roles. (**b**) A schema of CD137L function through direct or reverse signalling.

Here, we comprehensively investigated p53-regulated genes in calvarial bone. We found several p53-regulated pathways from comprehensive set of p53 targets and identified *CD137L*, *CDC42BPG*, and *FST* as novel direct p53 targets. Of these three genes, *CD137L* was considered a bone-specific p53 target that mediates growth suppression and immune response of osteosarcoma cells (Fig. 6a).

As we expected, pathway analysis showed that apoptosis-related genes were enriched in the p53-induced genes. Conversely, cell cycle-related genes were enriched in the p53-repressed genes. Numerous studies have revealed the role of canonical p53-mediated apoptosis and cell cycle arrest in tumour suppression^1, 2^. In bone or osteoblasts, p53-driven apoptotic response or the cell cycle arrest induced by acute DNA damage presumably contribute to the tumour-suppressive roles of p53 (Fig. 6a). Moreover, the cytokine-cytokine receptor interaction pathway may be related to the tumour-suppressive roles of p53 in bone according to our study. *Inhbb*, *Tnfrsf10b*, *Gdf5*, *Eda2r*, *Fas* and *Cd137l* were included in the cytokine-cytokine receptor interaction. Of these genes, *Tnfrsf10b*, *Eda2r* and *Fas* were reported to be p53 target genes and tumour-suppressor genes^27–30^.

*CD137L* was identified as a novel bone-specific p53 target gene. *CD137L* (also known as *tumour necrosis factor superfamily 9* [*TNFSF9*] or *4-1BBL*) is a member of the TNF superfamily, and the CD137L receptor/ligand system is used by antigen-presenting cells to costimulate T cell activity^26^. The activation of T cells plays a crucial role in the antitumour immune response, and cancer immunotherapy has now been clinically validated as an effective treatment^28^. To activate naive T cells, two key signals through major histocompatibility complex (MHC) class I/T cell receptor and costimulatory molecules are required^26^. The amplitude and quality of the T cell response are regulated by a balance between costimulatory and coinhibitory signals^26, 31^. p53 can regulate the T cell response by activating the expression of MHC class I molecules^32^ and suppressing the expression of coinhibitory molecules, such as PDL1^33^. This is the first report that shows p53 regulates costimulatory molecules, and our results emphasize the importance of p53 in immune regulation.

CD137L seems to have two roles in the suppression of osteosarcomagenesis (Fig. 6b). First, reverse CD137L signalling showed a growth-suppressive effect on osteoblasts and SaOS2 cells. CD137L reverse signalling was reported to induce apoptosis via the intrinsic pathway and cell cycle arrest^34^, and this was considered as a major mechanism of CD137L mediated growth suppression *in vitro*. T cells were shown to express CD137^26^. We also found that DNA damage induced *CD137* expression in osteoblasts or U2OS cells (Supplementary Fig. 6a,b). Thus, these cells may contribute to the induction of reverse CD137L signalling and suppress the tumour growth of osteosarcoma or osteosarcoma-initiating cells. However, many p53-regulated genes have a growth suppressive effect^1^, and the sole effect of *CD137L* on cell proliferation seems to be small.

Another function of CD137L in the suppression of osteosarcomagenesis is the activation of the T cell response. Recombinant Fc-fusion Cd137l protein activated the T-cells *in vitro*, and this data implies that direct CD137L signalling can mediate the osteosarcoma microenvironment and exert an anti-tumour immune effect through the activation of T cells. Mice inoculated with hepatocellular carcinoma cells expressing CD137L have been reported to develop a strong cytotoxic T lymphocyte response and long-term immunity against tumours^35^, and this data supports our hypothesis. Among 24 mouse tissues, induction of Cd137l was only found in bone, and these data indicates the importance of CD137L in osteosarcomagenesis. However, the mechanism how p53 induce Cd137l only in bone tissue is remained to be elucidated. Screening of Remap database indicates a binding of RUNX1 and RUNX3 that are essential to bone differentiation^36, 37^ to CD137L locus. Our RNA sequence data showed that Runx1 and Runx3 are highly expressed in the bone among 24 tissues^19^. In addition, RUNX family was shown to interact with p53 in DNA damage response^38^. Therefore, RUNX family would be co-factors for bone specific induction of Cd137l.

Our Microarray analysis using MCF10A and HCT116 cells indicated that *CD137L* mRNA expression was remarkably induced by ADR in MCF10A *p53* ^+/+^ and HCT116 *p53* ^+/+^ cells, although *CD137L* is also induced in MCF10A *p53* ^−/−^ and HCT116 *p53* ^+/+^ cells (Supplementary Fig. 7). On the other hand, CD137L was not induced in H1299 lung cancer cells by ectopic expression of p53. These result suggested that human *CD137L* expression was regulated by DNA damage in various types of cells through both p53-dependent and p53-independent manner. Difference in induction pattern between human CD137L and mouse Cd137l might be partially explained by the fact that human CD137L has four p53-binding elements that are not conserved in mouse Cd137l (Supplementary Fig. 8). In our study, CD137L-Fc showed an anti-tumour effect *in vivo*. Although further studies are needed to reveal the mechanism of this effect, CD137L/CD137 signalling could potentially be an actionable novel therapeutic target for osteosarcoma and other malignancies. One possible explanation of this effect is the T cell activation by CD137L-Fc. The ability of CD137L to potentiate strong and durable immune effector responses has made CD137L a clinically viable target for cancer immunotherapy for several types of cancer^39^.

We found that *CDC42BPG* and *FST* were novel p53 targets that also had growth-suppressive effects on U2OS and SaOS2 cells. Aside from the growth suppressive effect, CDC42BPG may exert an anti-tumour effect by inhibiting CDC42, which activates migration and metastasis in osteosarcoma cell lines^40, 41^. p53 was shown to promote apoptosis through activation of Cdc42/JNK1 pathway^42^ and CDC42 also negatively regulate p53 through miR-29^43^. CDC42BPG may be related to the p53/CDC42 feedback loop by inhibiting CDC42. FST may have a tumour-suppressor role via the inhibition of BMP2 activity^44^. BMP2 contributes to osteosarcoma growth^45^.

In conclusion, our transcriptome analysis revealed comprehensive p53-regulated pathways and genes in bone. We also uncovered the role of CD137L in osteosarcomagenesis and its potential therapeutic application. This study provides new insights into the roles of p53 in osteosarcomagenesis and the regulation of the immune response thorough p53.

## Materials and Methods

### Cell culture and transfection

U2OS and SaOS2 cell lines were purchased from the American Type Culture Collection (Manassas, VA, USA). LM8 and HEK293 cell lines were purchased from Riken BRC (Tsukuba, Ibaraki, Japan). The FreeStyle 293-F cell line was purchased from Thermo Fisher Scientific (Waltham, MA, USA). U2OS and SaOS2 are human osteosarcoma cell lines. LM8 is a murine osteosarcoma cell line. Primary osteoblasts were isolated from the calvarial bone of newborn mice as previously described^23^.

Cells were transfected with siRNA oligonucleotides, commercially synthesized by Sigma Genosys (Woodlands, TX, USA), using Lipofectamine RNAiMAX reagent (Invitrogen, Carlsbad, CA, USA). The sequences of the siRNA oligonucleotides are shown in Supplementary Table 2. All methods were carried out accordance with the Guidelines of the Institute of Medical Science, University of Tokyo.

### RNA sequencing

p53^−/−^ mice were provided by the RIKEN BioResource Center. Genotypes were confirmed by PCR analysis. All mice were maintained under specific pathogen-free conditions. Institute of Medical Science, University of Tokyo committee approved the experimental protocols and all experiments were performed in accordance with the Guidelines for Animal Experiments of the Institute of Medical Science, University of Tokyo. We used 4 groups of mice: K, W, KX and WX (n = 3 per group). p53^+/+^ and p53^−/−^ mice were X-ray-irradiated using a MBR-1520R-3 system (Hitachi). The calvarial bone and 23 additional tissues (thymus, heart, lung, kidney, spleen, liver, bladder, esophagus, stomach, colon, small intestine, testis, parorchis, seminal vesicle, muscle, bone marrow, tongue, eye ball, cerebrum, uterus, cartilage, ovary, mammary gland) were extracted at 24 h after exposure to 10 Gy radiation. One-week-old mice were used to obtain bone and cartilage tissues, and 10 week female mice were used for breast, uterus, and ovary. Six-week-old mice were used to obtain the other 19 tissues. Tissues were maintained in RNAlater stabilization solution at 4 °C until RNA collection. RNA was collected using an RNeasy Plus Universal Mini Kit (Qiagen, Valencia, CA, USA) after crushing the tissues with a Precellys homogenizer in QIAzol reagent. Quality was measured using a Bioanalyzer instrument (Agilent Technologies, Santa Clara, CA, USA). One microgram of RNA was subjected to polyA-tailed cDNA library construction using a TruSeq RNA Sample Preparation Kit v2 (Illumina, San Diego, CA, USA). Sequencing was performed in a Riken on an Illumina HiSeq. 2500 system according to a standard paired-end 101 bp protocol.

### RNA sequencing data analysis

We processed raw RNA sequencing data using a TopHat-Cufflinks pipeline. Before data processing, we checked the quality of data with FastQC. To quantify the expression levels of the genes and transcripts for all samples, 101-bp paired-end reads were aligned to the mouse reference genome mm9/GRCm37 by TopHat (v2.0.9). The mapping parameters follow the default setting in TopHat. After the read mapping, transcript and gene expression levels, which were represented by FPKM values, were calculated by Cufflinks (v2.2.1). Before we analysed the FPKM values in each of the tissues, 0.0001 was added to all the data to erase 0.

The criteria for p53-induced genes were as follows: (1) median FPKM value of WX/maximum FPKM value in median K, median KX or median W > 2, (2) t-test (WX vs. K, KX and W), P < 0.05, and (3) minimum FPKM value of WX > 1. The criteria for p53-repressed genes were (1) median FPKM value of WX/minimum FPKM value of median K, median KX or median W < 1/2, (2) t-test (WX vs. K, KX and W), P < 0.05, and (3) minimum FPKM value of W, K or KX > 1.

The Database for Annotation, Visualization, and Integrated Discovery (DAVID) was used for pathway analysis^46, 47^.

### cDNA microarray and its data processing

Gene expression analysis was performed using a SurePrint G3 Human GE 8 × 60 K microarray (Agilent, Santa Clara) according to the manufacturer’s protocol. Briefly, MCF10A *p53* ^+/+^, MCF10A *p53* ^−/−^, HCT *p53* ^+/+^ and HCT *p53* ^−/−^cells were treated with 2 µg/ml of adriamycin for 2 h and incubated at 37 °C until harvest. At 0 h, 12 h, 24 h and 48 h after ADR treatment, total RNA was isolated from the cells using standard protocols. Each RNA sample was labeled and hybridized to array slides. These data were shown in our previous studies^48, 49^. The MCF10A microarray data is available from the NCBI GEO database (GSE98727).

### Quantitative real-time PCR

qPCR was conducted using SYBR Green I Master on a Light Cycler 480 (Roche, Basel, Switzerland). Primers sequences are indicated in Supplementary Table 2.

### Gene reporter assay

DNA fragments, including the potential p53BS of each gene, were amplified and subcloned into pGL4.24 promoter vectors (Promega, Fitchburg, WI, USA). A reporter assay was performed using a Dual-Luciferase assay system (Promega) as previously described^50^. We used SaOS2 cells (p53 null) for a reporter assay.

### Chromatin immunoprecipitation assay

ChIP assay was performed using an EZ-Magna ChIP G Chromatin Immunoprecipitation kit (Merck Millipore, Darmstadt, Germany) following the manufacturer’s protocol. Before immunoprecipitation, 1% of the supernatant was removed as “input”. Column-purified DNA was quantified by qPCR.

### Western blotting

Western blotting was performed according to standard protocols. Anti-β-actin monoclonal antibody (clone AC15) was purchased from Sigma-Aldrich (St. Louis, MO, USA). Anti-p53 monoclonal antibody (clone DO-7) and anti-p21 monoclonal antibody (clone EA10) were purchased from Merck Millipore. Anti-HA monoclonal antibody (clone 3F10) was purchased from Roche. Human anti-CD137L monoclonal antibody (EPR1172Y) was purchased from GeneTex (Irvine, CA, USA). Mouse anti-Cd137l monoclonal antibody (sc-11819) was purchased from Santa Cruz (Dallas, TX, USA). α-Tubulin antibody (11H10) was purchased from Cell Signaling Technology (Danvers, MA, USA).

### Immunohistochemistry

The calvarial bone was removed from 1-week-old *p53* ^+/+^ or *p53* ^−/−^ mice. We used four groups of mice: K, W, KX and WX. Each group contained 3 mice. Goat anti-Cd137l antibody (sc-11819, Santa Cruz) and rat anti-Ki67 antibody (clone MIB-5, Agilent Technologies) were added to each slide after blocking of the endogenous peroxidase and proteins. Staining was evaluated by two independent investigators.

### Colony formation assay

A colony formation assay was performed in six-well culture plates. Cells were transfected with pCAGGS/CD137L, pCAGGS/CDC42BPG, pCAGGS/FST or mock plasmid using FuGENE6 (Roche). Cells were cultured in the presence of Geneticin (1.0 mg/ml, 1.0 mg/ml or 1.2 mg/ml for U2OS, SaOS2 or LM8 cells, respectively) (Thermo Fisher Scientific) for 1-2 weeks. Colonies were stained with crystal violet (Sigma-Aldrich) and quantified using ImageJ software.

### Cell proliferation assays for osteosarcoma cell lines and osteoblasts

Cell proliferation was assessed using a CellTiter-Glo Luminescent Cell Viability Assay (Promega) according to the manufacturer’s protocol. Briefly, SaOS2 cells or osteoblasts were seeded at 4.0 × 10^3^ or 7.5 × 10^3^ cells/100 μl of medium/well, respectively, in 96-well culture plates that had been coated with CD137-Fc or mock-Fc. After culture for 48 h, the absorbance values were measured using a microplate reader (Biotek Instruments, Winooski, VT, USA).

### Construction of stable cell lines and in vivo studies

LM8 cells were transfected using Lipofectamine 2000 reagent (Invitrogen) with the mock or Cd137l-pCAGGS expression vector, both of which had the *neo* gene to resist Geneticin. These clones were placed for 3 weeks in culture medium containing 1.0 mg/ml Geneticin to select for resistant cells. For each vector, 3 clones were selected, and we quantified Cd137l expression using western blotting and immunohistochemistry.

Six-week-old male C3H/HeJ mice were subcutaneously injected in the left and right flanks with a 0.1 ml phosphate buffered saline (PBS) containing 1 × 10^6^ LM8 cells expressing Cd137l (Cd137l-1, Cd137l-2, Cd137l-3) or mock (mock 1, mock2, mock3). Each group contained 2 mice. The tumour volume was measured every 2 or 3 days and was calculated using the following formula: (long diameter) × (short diameter)^2^ × 0.52. The results are expressed as the mean ± standard deviation (SD). Statistical significance was determined by the Wilcoxon rank-sum test. Institute of Medical Science, University of Tokyo committee approved the experimental protocols and all experiments were performed in accordance with the Guidelines for Animal Experiments of the Institute of Medical Science, University of Tokyo.

### Construction of human CD137-Fc, mouse Cd137-Fc and mouse Cd137l-Fc

The extracellular coding sequences of CD137 (human, amino acids 24–186; mouse, amino acids 24–187) or Cd137l (mouse, amino acids 104–304) and the Fc region of human IgG (amino acids 100–329) were amplified and subcloned into a pCAGGS expression plasmid according to previous reports^51, 52^. FreeStyle 293-F cells were transfected using FuGENE6 according to the manufacturer’s protocol. Recombinant proteins (human CD137-Fc, mouse Cd137-Fc and mouse Cd137l-Fc) were extracted from the culture media by affinity purification using protein A-Sepharose (Invitrogen), and it was purified using a dialysis cassette (0.1–0.5 ml, 10 K Molecular weight cut off, Thermo scientific). Immunocytochemistry was performed using a human IgG antibody (American Qualex International, San Clemente, CA, USA).

### In vitro activity assay of mouse Cd137l-Fc for T cells

The effects of Cd137l-Fc on T cells were investigated by sandwich ELISA measurement of IL-2 production and a cell proliferation assay. T cells were isolated from splenocytes which were removed from 6-week-old male C3H/HeJ mice (CLEA Japan, Tokyo, Japan). CD4^+^ and CD8^+^ cells were sorted using FACS Aria (BD Biosciences, San Jose, CA, USA). CD4^+^ and CD8^+^ cells were seeded at 5.0 × 10^5^ or 2.5 × 10^5^ cells/500 μl medium/well, respectively, in 24-well culture plates precoated with 5.0 μg/ml anti-CD3 antibody (clone 145-11 C, Biolegend) in the presence of 20 μg/ml Cd137l-Fc or 20 μg/ml mock-Fc. Anti-CD28 antibody (clone 37.51, eBioscience, San Diego, CA, USA, 1.5 μg/ml) was used as a positive control. After 48 h, IL-2 production was determined by ELISA (mouse IL2 DuoSet ELISA, R and D systems, Minneapolis, MN, USA) for the above culture supernatants. All assays were performed in triplicate.

### Effect of Cd137-Fc on tumour growth in vivo

Six-week-old male C3H/HeJ mice were subcutaneously injected in the left flank with 0.1 ml of PBS containing 1 × 10^6^ LM8 cells. When tumours reached 0.5 cm in diameter, at approximately the 7th day after tumour implantation, each group of mice (n = 3) was treated with 0.2 ml (50 µg) of Cd137l-Fc fusion protein or control mock-Fc by intraperitoneal injection for 3 days. Statistical significance was determined by the Wilcoxon rank-sum test. Institute of Medical Science, University of Tokyo committee approved the experimental protocols and all experiments were performed in accordance with the Guidelines for Animal Experiments of the Institute of Medical Science, University of Tokyo.

## Electronic supplementary material

Supplementary Figure 1-9, Supplementary Table 1 and 2
