# Supplementary material for: Identification of a p53 target, CD137L, that mediates growth suppression and immune response of osteosarcoma cells
Source: Sci Rep. 2017 Sep 6;7:10739. doi: 10.1038/s41598-017-11208-x (PMC5587585; doi:10.1038/s41598-017-11208-x)
Supplement: Supplementary file 1 — Supplementary Figure 1-9, Supplementary Table 1 and 2 [file 41598_2017_11208_MOESM1_ESM.pdf]

Identification of a p53 target, *CD137L*, that mediates growth suppression and immune response of osteosarcoma cells

Yusuke Tsuda, Chizu Tanikawa, Takafumi Miyamoto, Makoto Hirata, Varalee Yodsurang, Yao-zhong Zhang, Seiya Imoto, Rui Yamaguchi, Satoru Miyano, Hiroshi Takayanagi, Hirotaka Kawano, Hidewaki Nakagawa, Sakae Tanaka, Koichi Matsuda

Supplementary Figure 1.

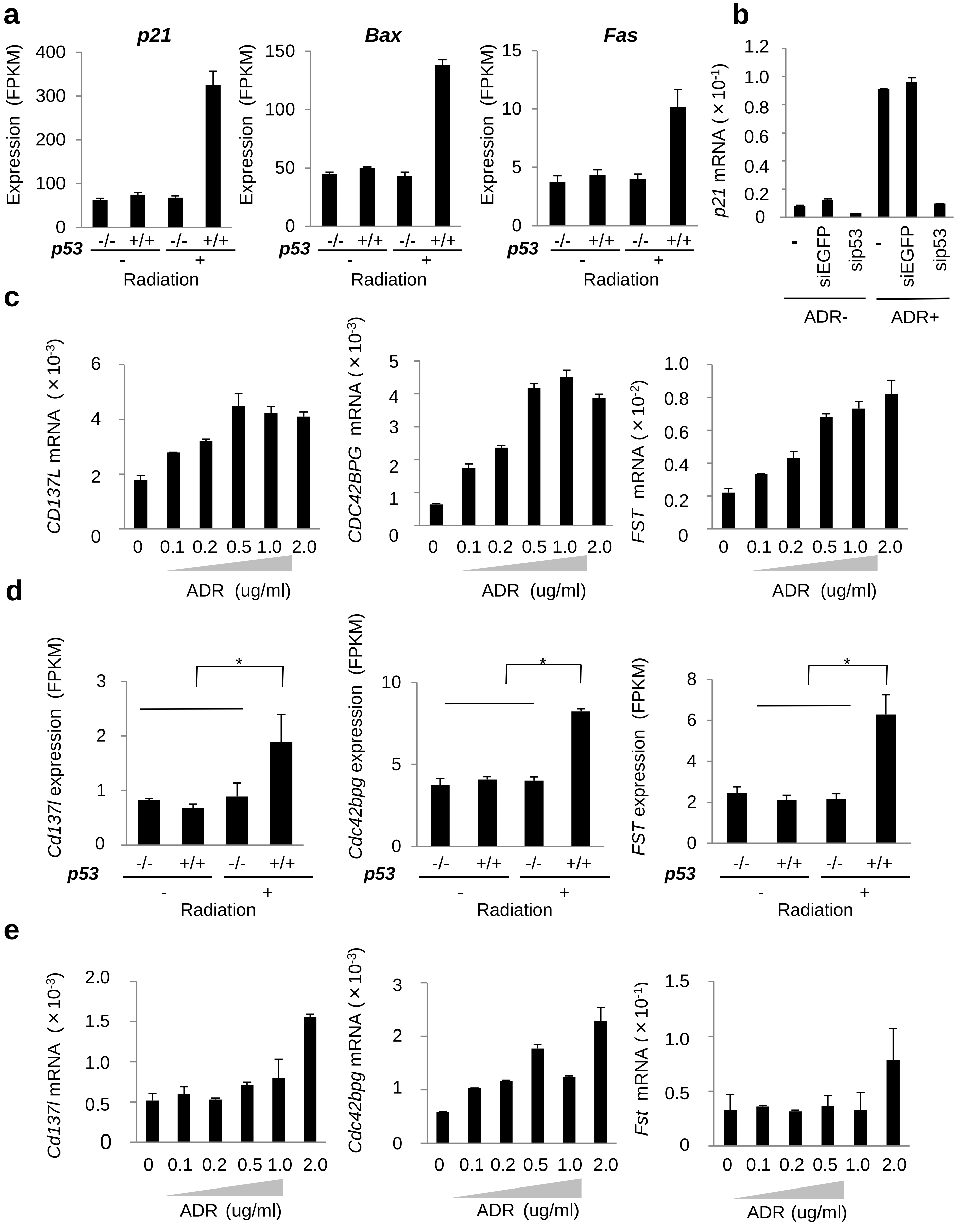

Supplementary Figure 1. Regulation of *CD137L*, *CDC42BPG* and *FST* by p53

(a) Expression of representative p53 target genes *p21*, *Bax* and *Fas* based on RNA sequencing data. Error bars represent SD (n = 3). (b) At 24 h after transfection with each siRNA, U2OS cells were treated with ADR (2 μg/ml for 2 h). Then, 36 h after treatment, qPCR was performed. siRNA against *EGFP* was used as a control. β-actin was used for normalization of the expression levels. Error bars represent SD (n = 2). (c) qPCR in U2OS cells harvested 36 h after ADR treatment (0-2 μg/ml for 2 h). β-actin was used for normalization of the expression levels. Error bars represent SD (n = 2). (d) *Cd137l*, *CDC42BPG* or *FST* expression from RNA sequencing data. FPKM, fragments per kilobase of exon per million mapped fragments. Error bars represent SD (n = 3). *$P < 0.05$, Student's t-test. (e) qPCR in $p53^{+/+}$ calvarial osteoblasts harvested 36 h after ADR treatment (2 μg/ml for 2 h). β-actin was used for normalization of the expression levels. Error bars represent SD (n = 3).

Supplementary Figure 2.

a

Human

Consensus	RRRCWWGYYYRRRCWWGYYY
CD137L-BS-A	AcACTAGCCagtAACATtaCT
CD137L-BS-B	GGGCAcGgTaAcGCATGaCT

Mouse

Consensus	RRRCWWGYYYRRRCWWGYYY
Cd137l-BS	GtGCATGCaCAtACATGCag

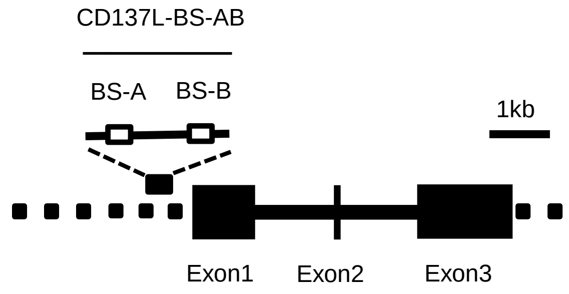

b

Human

Consensus	RRRCWWGYYYRRRCWWGYYY
CDC42BPG-BS-A	AGGCATGagCcAcCATGCCC
CDC42BPG-BS-B	AtGCcAGTCCcAGcCcTGCCC
CDC42BPG-BS-C	ctGCTTGTCTctACTTGTCC

Mouse

Consensus	RRRCWWGYYYRRRCWWGYYY
Cdc42bpg-BS	cAACTTGTCCctGctCcTGCCC

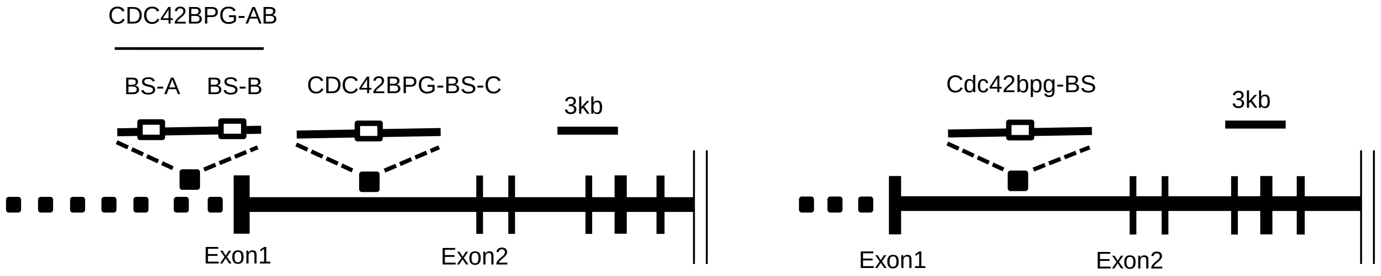

c

Human

Consensus	RRRCWWGYYYRRRCWWGYYY
FST-BS1	GAACcAGTaTtGACcTGTgT
FST-BS2	GGACcTGCCCAGACATGTCC

Mouse

Consensus	RRRCWWGYYYRRRCWWGYYY
Fst-BS	cGACATGTaaAAACATcCgT

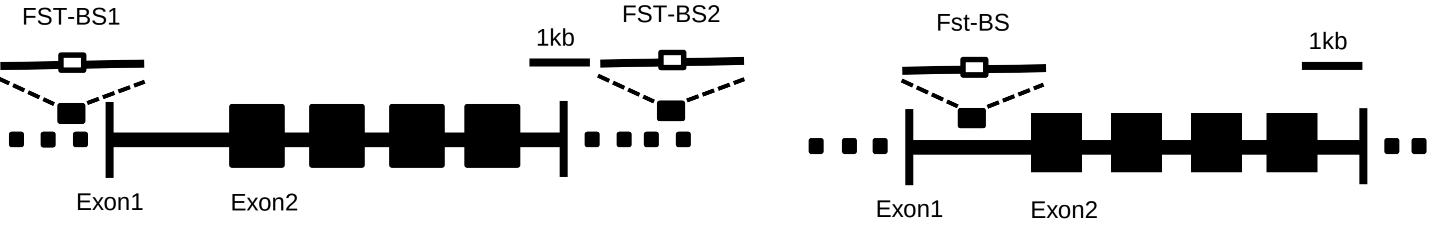

Supplementary Figure 2. Genomic structures
(a)(b)(c) The genomic structures of the human (left) and mouse (mouse) *CD137L* (a), *CDC42BPG* (b) and *FST* (c) genes. White boxes represent the location of potential p53-binding sequences (p53BSs). R, purine; W, A or T; Y, pyrimidine. Nucleotides identical to the consensus sequence are written in capital letters.

Supplementary Figure 3.

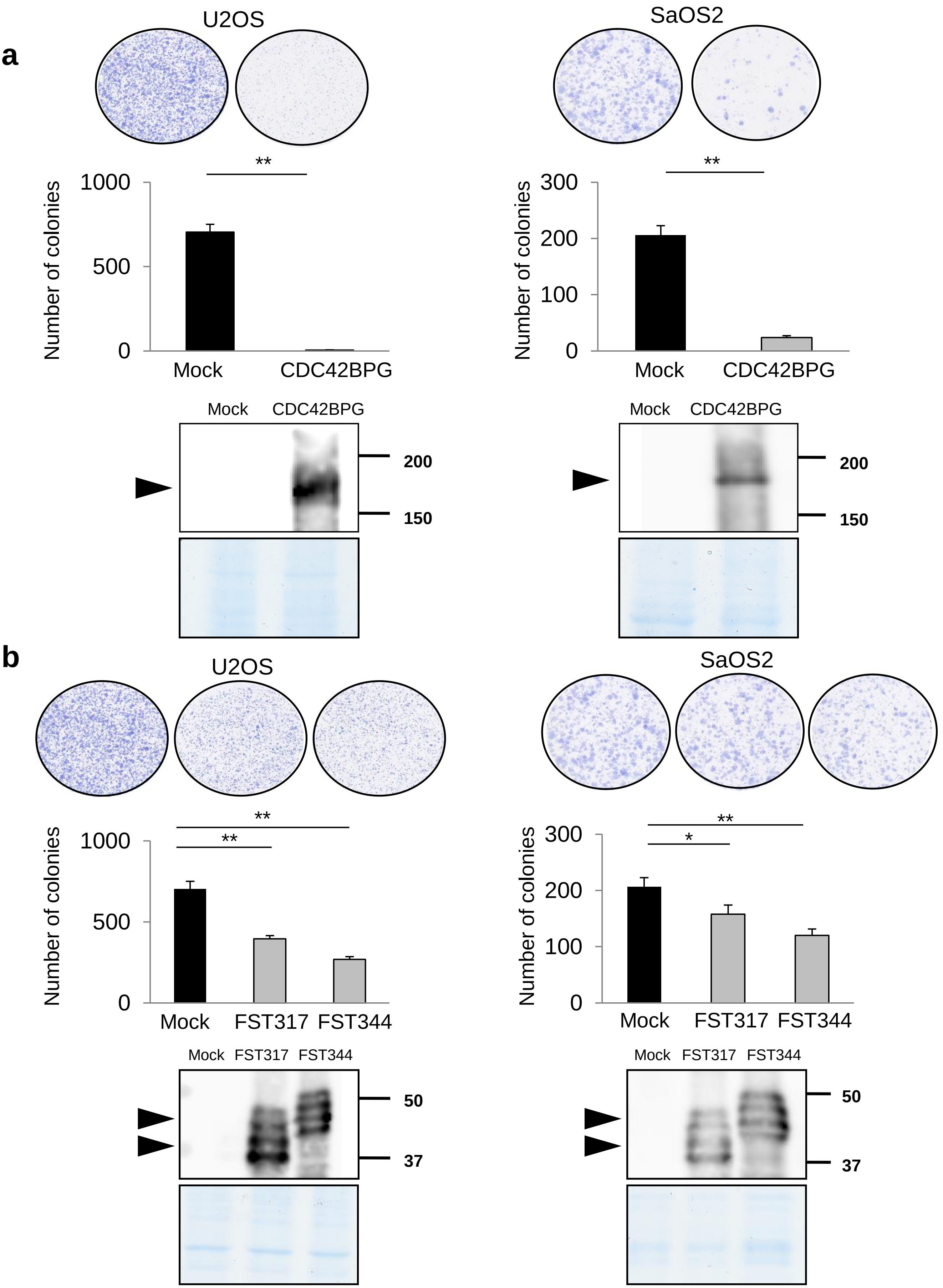

Supplementary Figure 3. Colony formation assay

(a)(b) A colony formation assay was performed. After ectopic expression of *CDC42BPG* (a), *FST* (b) or mock, the number of U2OS (left) or SaOS2 (right) colonies was determined. FST317 and FST344 are isoforms of FST. Whole cell extracts were subjected to western blotting with an anti-HA antibody. Coomassie Brilliant Blue staining was used as loading control.

Supplementary Figure 4.

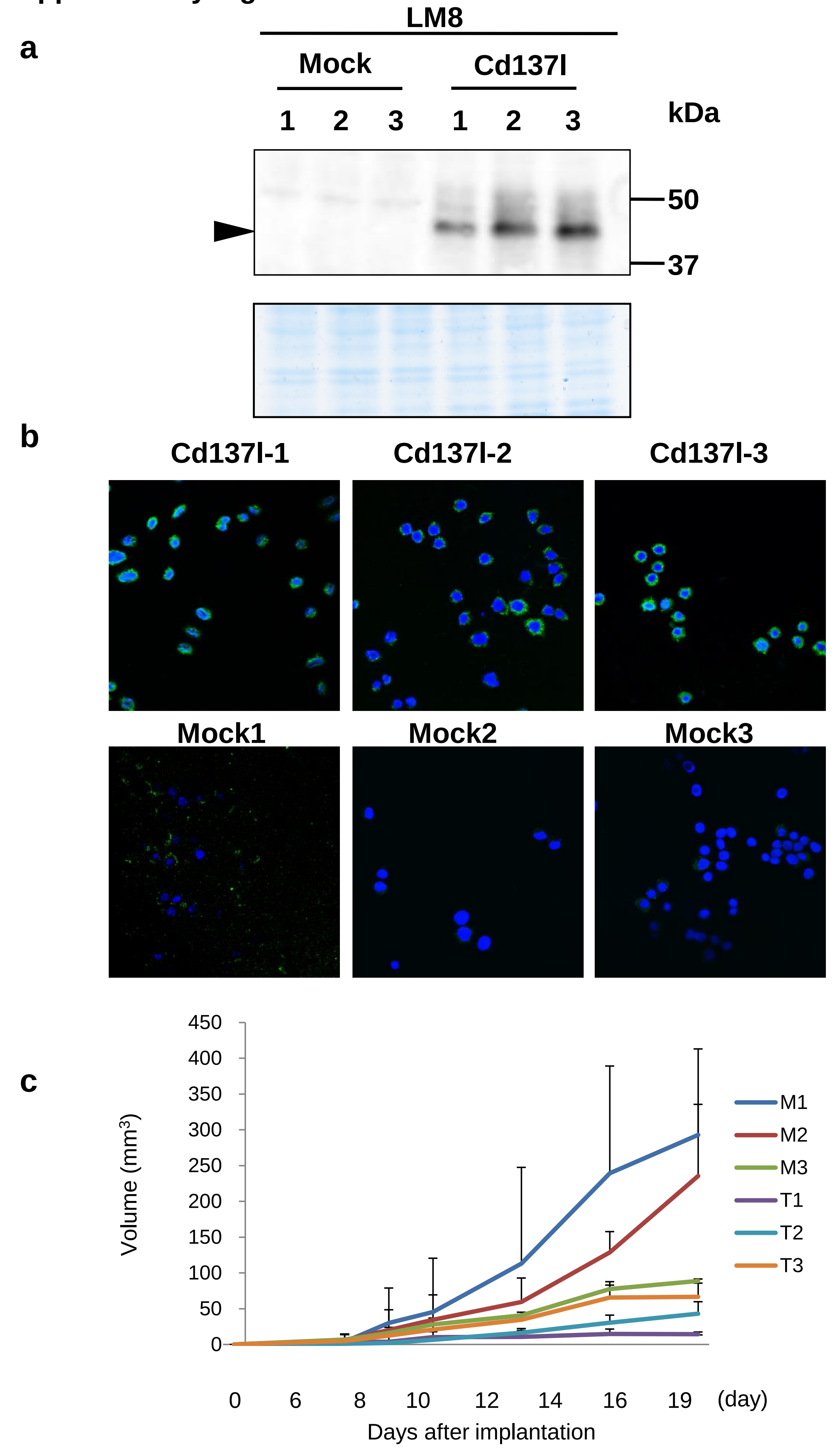

Supplementary Figure 4. Construction of stable cell lines and in vivo studies
(a) At 24 h after seeding, whole cell extracts were subjected to western blotting with an anti-Cd137l antibody. Coomassie Brilliant Blue staining was used as loading control. (b) Immunohistochemical staining for HA protein in Cd137l-expressing cells. (c) Cd137l (n = 3) or mock (n = 3) stable expression cell lines were inoculated into the left and right flanks of C3H mice; each group contains 2 mice. Tumor volume was calculated every 2 or 3 days.

Supplementary Figure 5.

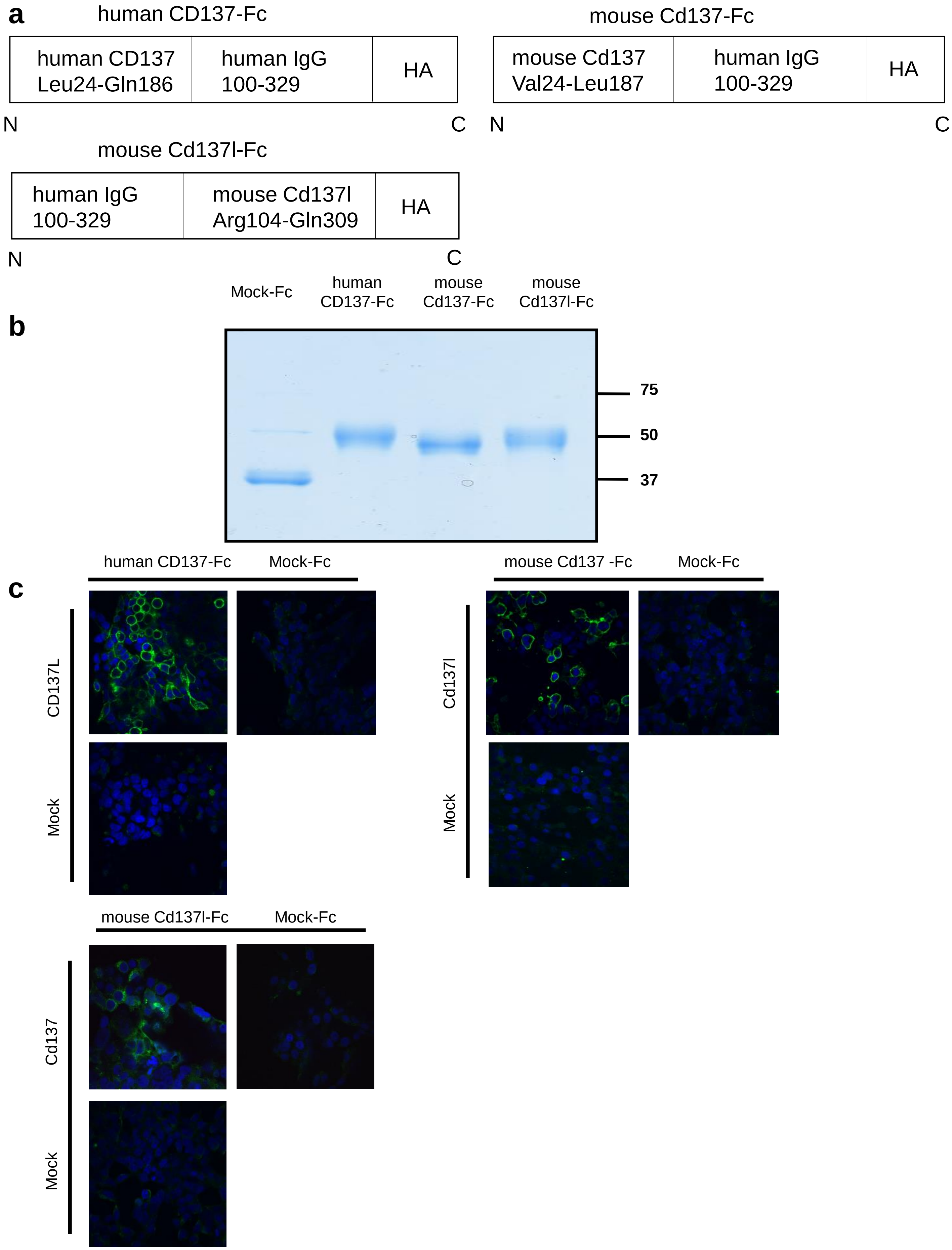

Supplementary Figure 5. Construction of recombinant proteins and in vivo studies
(a) The structures of recombinant proteins fused with the Fc fragment of IgG. Leu, Leucine; Gln, Glutamine; Val, Valine; Arg, Arginine. (b) Coomassie Brilliant Blue staining of recombinant proteins after purification. (c) After transfection with plasmid expressing CD137L or mock, the HEK293 cells were incubated with CD137-Fc (2 μg/ml) or mock-Fc (2 μg/ml) for 2 h. Immunohistochemical staining was performed using human IgG antibody to confirm binding ability in each recombinant protein. We also confirmed the interaction between Cd137 and Cd137l-Fc.

Supplementary Figure 6.

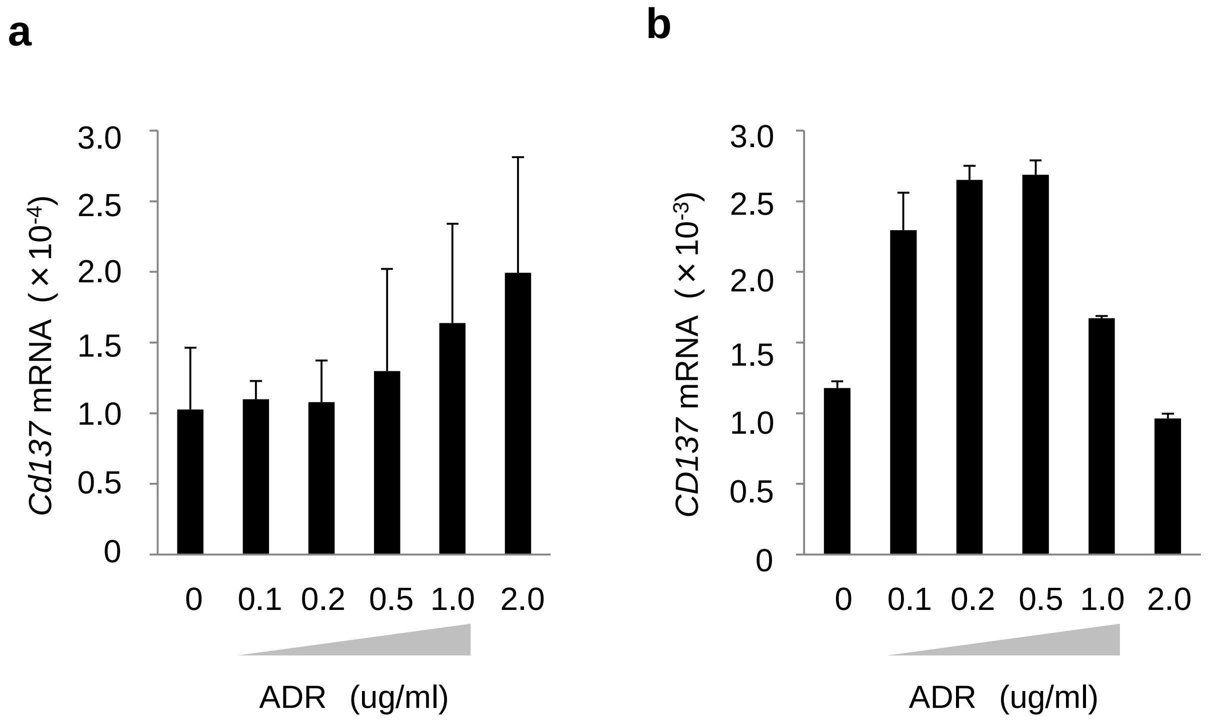

Supplementary Figure 6. Expression of CD137
(a) qPCR of p53$^{+/+}$ osteoblasts harvested 36 h after ADR treatment (0-2 µg/ml for 2 h). β-actin was used for normalization of the expression levels. Error bars represent SD (n = 2). (b) qPCR of U2OS cells harvested 36 h after ADR treatment (0-2 µg/ml for 2 h). β-actin was used for normalization of the expression levels. Error bars represent SD (n = 2).

Supplementary Figure 7.

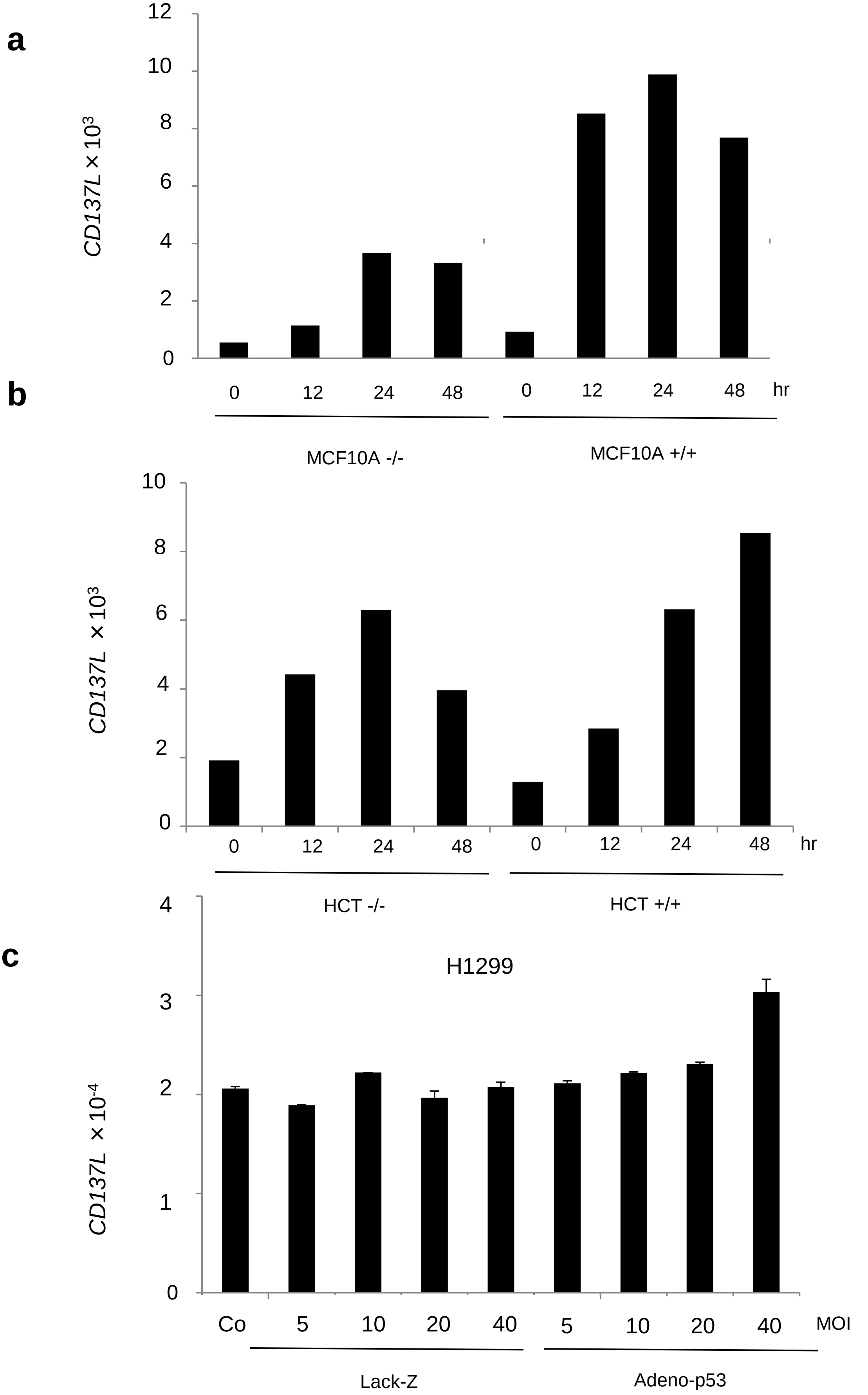

Supplementary Figure 7. Expression of CD137

(a) (b) Microarray analysis was performed after treatment with 2 µg/ml of adriamycin for 2. At 0 h, 12 h, 24 h and 48 h after ADR treatment, RNA was collected. (c) qPCR was performed after Lack-Z or Adeno-p53 infection (5-40MOI). β-actin was used for normalization of the expression levels. Error bars represent SD (n = 2).

Supplementary Figure 8.

p53 binding regions in the *CD137L* gene (+/- 1kb)
Ch19: 6530010-6536939 (GRCh37/hg19)

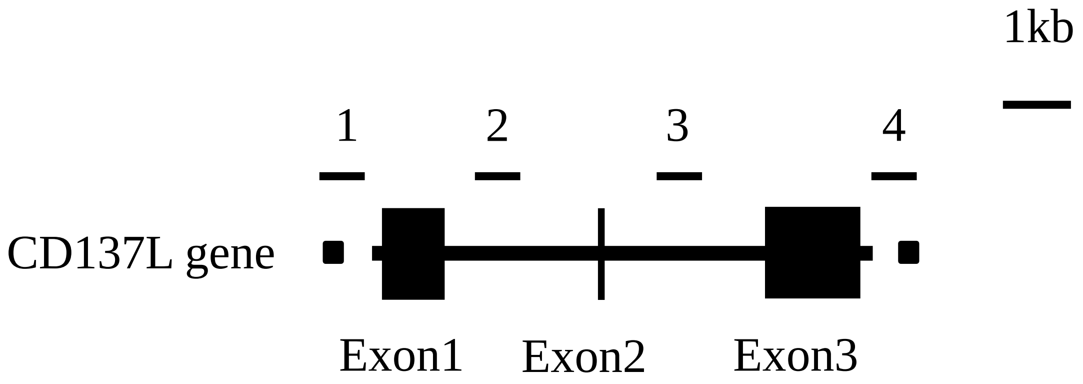

1	chr19	Promoter	6530921	6531665
2	chr19	Intron1	6532281	6532324
3	chr19	Intron2	6534452	6534634
4	chr19	Downstream	6536671	6536767

Supplementary Figure 8. Remap database indicates four p53 binding regions in CD137L gene (within ±1kb, GRCh37/hg19)

Supplementary Figure 9.

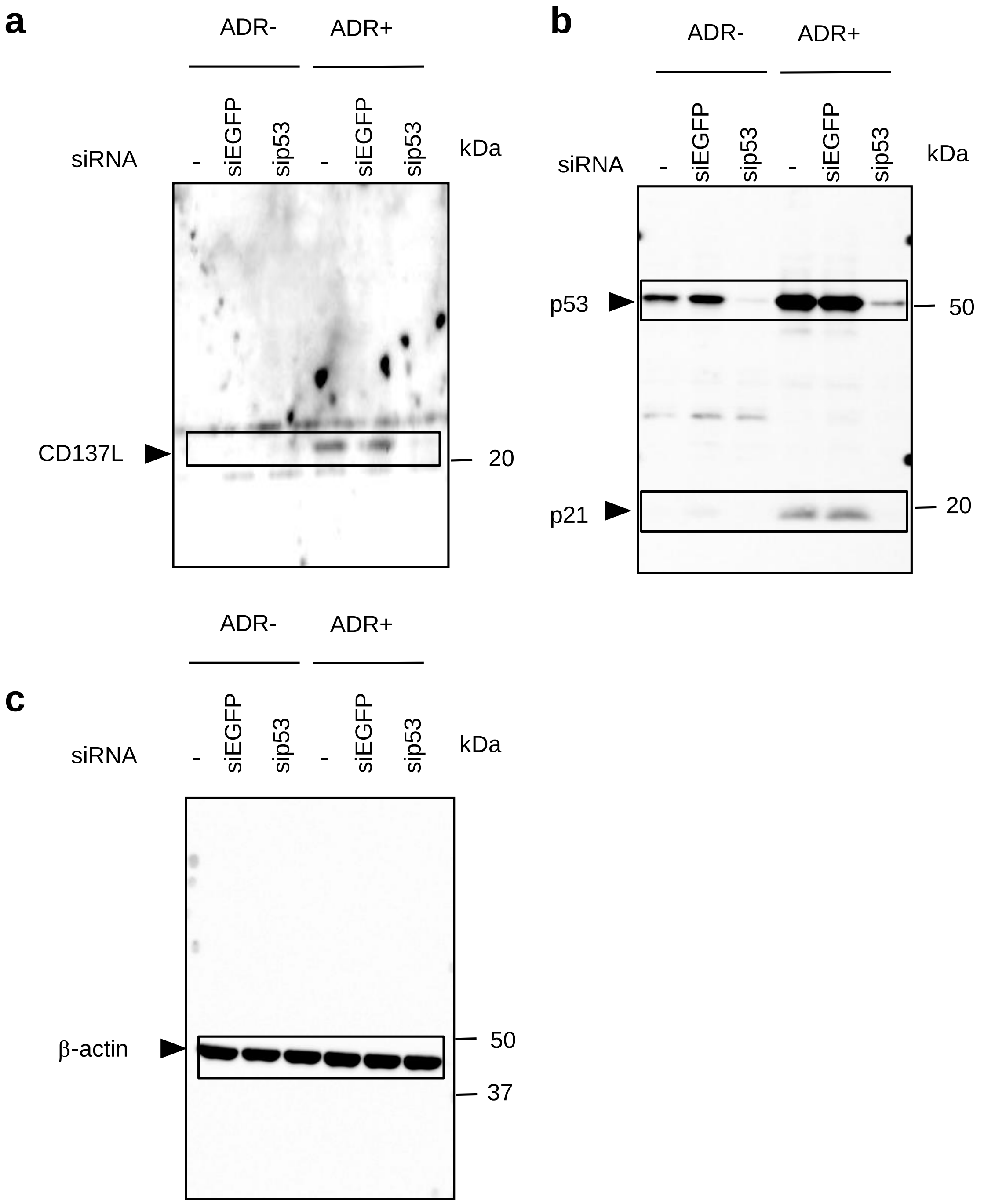

Supplementary Figure 9. Full-length gels and blots are shown in Figure 3b

Supplementary Table 1a. p53-induced gene list

gene	FPKM value					status
	WX	KX	W	K	WX/max	
Aen	35.15	7.68	8.84	7.32	3.98	R
AI414108	1.57	0.36	0.76	0.51	2.07	M
Ak1	79.91	32.19	32.61	33.52	2.38	R
Ano3	1.37	0.05	0.03	0.06	24.64	N
Anxa8	36.83	16.46	15.49	14.13	2.24	N
Ass1	11.15	3.46	2.28	2.63	3.22	N
Atp1a2	13.71	6.47	3.39	4.68	2.12	N
B230120H23Rik	8.86	4.11	4.01	3.88	2.16	M
Bax	139.69	42.14	50.40	43.63	2.77	R
Bbc3	16.85	4.86	5.52	4.87	3.05	R
Cbln2	1.27	0.41	0.63	0.50	2.02	N
Ccng1	75.24	14.88	17.13	15.05	4.39	R
Cd80	2.00	0.64	0.51	0.41	3.14	N
Cd137l	1.76	0.81	0.72	0.81	2.17	N
Cdc42bpg	8.31	4.05	3.97	3.88	2.05	N
Cdkn1a	343.52	66.17	74.00	64.30	4.64	R
Celf5	5.47	0.94	1.68	1.14	3.25	N
Ces2e	2.25	0.63	0.63	0.54	3.57	M
Cox6b2	48.06	8.77	7.36	8.15	5.48	N
Cpt1c	27.30	9.44	11.19	10.20	2.44	R
Dcxr	41.06	6.28	6.28	5.57	6.54	N
Dynlrb2	1.31	0.17	0.09	0.62	2.13	N
Eda2r	5.71	0.13	0.25	0.22	22.60	R
Ephx1	73.75	6.92	9.36	8.42	7.88	N
Exoc4	17.97	4.26	5.22	4.05	3.44	N
Fam212b	8.10	0.53	1.46	1.22	5.56	N
Fas	9.32	4.00	4.37	3.99	2.13	R
Foxj1	1.72	0.31	0.54	0.72	2.38	N
Fst	6.16	2.13	2.01	2.30	2.67	N
Gas6	364.76	106.60	122.06	121.24	2.99	N
Gdf15	3.07	0.27	0.25	0.49	6.31	R
Gdf5	1.52	0.41	0.53	0.71	2.14	N
Gfap	13.30	1.35	3.26	2.14	4.08	N
Gm11974	19.70	7.27	8.98	8.32	2.19	M
Gria3	9.27	2.07	2.76	2.47	3.35	N
Inhbb	4.18	1.23	1.90	1.65	2.20	N
Lyve1	11.05	3.20	3.49	4.15	2.67	N
Mdm2	32.38	15.10	15.44	14.19	2.10	R
Mgmt	19.86	6.86	6.28	6.37	2.90	R
Mrap	4.22	0.83	0.87	1.25	3.38	N
Perp	8.48	2.65	3.54	3.91	2.17	R
Phlda3	90.31	18.02	23.24	19.30	3.89	R
Pmaip1	8.73	2.88	2.39	1.86	3.03	R
Polk	4.79	1.81	1.89	1.87	2.53	R
Pqlc3	27.80	12.27	12.74	10.70	2.18	N
Psrc1	29.63	7.09	5.53	4.77	4.18	R
Ptprv	16.15	7.26	7.25	7.43	2.17	R
Pvt1	2.63	1.17	1.00	0.89	2.25	R
Rnf169	6.01	2.72	2.85	2.91	2.07	N
Rprm	3.03	0.67	0.92	0.84	3.29	R
Rps27l	222.32	95.94	103.78	96.33	2.14	R
Serpina3n	2.53	0.94	0.83	0.94	2.69	M
Sesn2	17.52	3.66	4.21	3.62	4.16	R
Slc19a2	7.75	1.19	1.73	1.35	4.49	R
Slc2a9	2.73	0.64	0.61	0.65	4.23	R
Slco1c1	3.56	0.46	0.77	0.84	4.25	N
Svop	1.44	0.01	0.39	0.23	3.67	N
Syna	1.24	0.48	0.31	0.33	2.61	M
Tnfrsf10b	9.93	2.95	3.04	2.91	3.27	R
Trim7	7.52	1.62	1.27	1.74	4.33	N
Trp53inp1	25.23	5.36	5.87	4.88	4.30	R
Upk1b	2.42	0.27	0.19	0.38	6.43	N
Vnn1	1.67	0.44	0.38	0.55	3.04	N
Zfp365	5.47	1.72	1.91	1.55	2.87	R
Zmat3	15.04	3.06	3.82	3.15	3.94	R
1700003M07Rik	1.32	0.37	0.59	0.51	2.25	M
1700007K13Rik	3.76	0.05	0.29	0.23	13.01	M
2010001M06Rik	10.09	1.03	2.00	1.84	5.06	M
9030617O03Rik	11.29	2.76	3.29	2.68	3.43	M

FPKM, fragments per kilobase of exon per million mapped fragments

WX: irradiated *p53*$^{+/+}$ group, KX: irradiated *p53*$^{-/-}$ mice group, W: non-irradiated *p53*$^{+/+}$ group, K: non-irradiated *p53*$^{-/-}$ group.

WX/max is median of WX / maximum value in median K, median KX or median W.

R, reported gene as p53 target , N, non-reported gene as p53 target, M, mouse genes which don’t have human homologu.

Supplementary Table1b. p53-repressed gene list

gene	FPKM value					status
	WX	KX	W	K	WX/max	
Ankle1	1.07	4.59	3.11	3.05	0.35	N
Anln	1.23	5.02	3.27	3.58	0.38	N
Apitd1	1.37	3.08	3.67	3.01	0.45	N
Asf1b	1.95	10.43	7.53	7.02	0.28	N
Atad2	1.34	3.99	3.02	3.06	0.44	N
Aurkb	2.27	14.10	7.80	9.87	0.29	N
BC030867	0.43	2.41	1.23	1.47	0.35	M
Birc5	6.25	35.08	20.28	22.11	0.31	K
Bub1	0.75	3.99	2.14	2.42	0.35	N
Bub1b	2.22	8.43	5.06	5.47	0.44	N
Ccna2	5.40	26.62	16.43	17.42	0.33	N
Ccnb2	5.54	27.70	12.98	14.19	0.43	N
Ccne2	0.65	1.92	1.87	1.74	0.38	N
Ccr2	0.79	1.94	2.02	2.53	0.41	N
Cd79b	1.01	12.18	6.23	4.27	0.24	N
Cdc45	1.91	7.22	4.69	5.22	0.41	N
Cdc6	0.39	2.05	1.53	1.61	0.25	N
Cdca2	0.94	4.08	2.23	2.27	0.42	N
Cdca3	5.72	30.56	20.39	19.34	0.30	N
Cdca5	0.92	5.25	2.75	3.56	0.34	N
Cdca7	2.14	6.02	5.26	5.33	0.41	N
Cdca7l	0.67	2.43	1.99	2.03	0.33	N
Cdca8	4.13	18.56	10.12	11.95	0.41	N
Cdk1	4.57	19.31	9.90	10.90	0.46	N
Cdkn3	2.15	9.79	5.10	4.55	0.47	N
Cenpe	0.75	2.77	1.54	1.70	0.49	N
Cenpf	0.44	2.37	1.40	1.41	0.31	N
Cenph	1.41	5.88	3.22	4.03	0.44	N
Cenpi	0.82	2.89	1.84	1.96	0.44	N
Cenpk	1.55	4.67	3.50	3.59	0.44	N
Cenpm	1.24	5.52	4.00	4.42	0.31	N
Cenpn	1.82	5.94	3.90	4.34	0.47	N
Cenpp	1.11	4.86	3.27	3.44	0.34	N
Cep55	1.49	7.26	3.43	4.40	0.44	N
Chad	17.37	35.66	40.02	38.51	0.49	N
Chek1	1.01	2.83	2.11	2.17	0.48	R
Chtf18	1.12	3.31	2.29	2.76	0.49	N
Ckap2l	2.82	10.37	6.32	6.79	0.45	N
Clspn	0.50	2.81	1.90	2.01	0.27	N
Cybb	1.09	3.62	2.38	3.11	0.46	N
Depdc1a	0.95	4.97	2.82	3.26	0.34	N
Dhfr	0.96	2.72	2.08	2.21	0.46	N
Dlgap5	1.62	7.76	3.63	3.91	0.45	N
Dna2	0.59	1.54	1.21	1.38	0.49	N
Dscc1	0.37	1.82	1.18	1.61	0.31	N
E2f2	0.58	3.07	1.85	1.62	0.36	N
E2f8	0.78	3.97	3.06	2.86	0.27	N
Ect2	0.92	3.71	2.08	2.29	0.44	N
Esco2	0.40	2.57	2.17	2.26	0.18	N
Fam64a	3.76	13.57	9.54	10.56	0.39	N
Fbxo5	1.89	7.56	4.81	5.33	0.39	N
Fignl1	1.17	4.28	3.18	3.46	0.37	N
Foxm1	2.40	8.85	6.00	6.30	0.40	N
Gins2	4.85	13.00	9.85	11.45	0.49	N
Gpha2	10.17	26.16	25.66	20.35	0.50	N
Gsg2	0.54	2.41	1.26	1.38	0.43	N
Hells	0.97	3.65	2.48	2.67	0.39	N
Hmgb2	12.97	43.96	37.27	32.87	0.39	N
Incenp	5.67	18.42	11.53	11.94	0.49	N
Iqgap3	0.56	3.02	2.28	2.48	0.25	N
Kif11	1.35	7.05	4.40	4.81	0.31	N
Kif15	0.46	2.98	1.97	2.06	0.23	N
Kif18a	0.65	2.05	1.60	1.37	0.47	N
Kif20b	0.52	2.16	1.24	1.39	0.42	N
Kif22	3.84	14.39	9.36	9.80	0.41	N
Kif23	1.72	7.99	4.79	5.09	0.36	N
Kif2c	1.86	8.10	3.95	4.83	0.47	N
Kif4	0.88	3.80	2.14	2.54	0.41	N
Lmnb1	7.19	23.94	17.09	18.85	0.42	N
Map4k1	0.57	2.28	1.34	1.18	0.49	N
Mcm10	0.79	3.44	1.94	2.11	0.41	N
Mcm2	4.28	14.37	10.15	11.26	0.42	N
Mcm3	5.06	18.28	12.70	13.30	0.40	N
Mcm5	3.50	15.25	9.45	10.67	0.37	N
Mcm6	8.75	24.29	19.22	20.86	0.46	N
Melk	0.80	5.19	3.27	3.90	0.24	N
Mis18bp1	0.64	2.94	1.78	1.99	0.36	N
Mki67	1.89	12.20	8.92	7.99	0.24	R
Mns1	1.56	4.96	3.26	3.37	0.48	N
Mxd3	1.12	7.81	6.11	6.10	0.18	N
Mybl2	1.00	4.55	2.89	3.23	0.35	N
Napsa	0.77	5.17	2.14	2.62	0.36	N
Ncapg	0.63	3.25	2.06	2.46	0.31	N
Ncapg2	0.88	2.60	1.89	2.16	0.47	N
Ncaph	3.19	9.88	6.55	6.85	0.49	N
Ndc80	1.20	6.96	4.64	4.76	0.26	N
Neil3	1.25	6.67	4.58	4.60	0.27	N
Nek2	1.10	5.17	2.44	2.43	0.45	N
Nsl1	0.84	2.93	1.94	1.91	0.44	N
Nuf2	1.77	9.61	5.89	6.39	0.30	N
Nusap1	2.54	13.80	7.75	7.80	0.33	N
Oip5	0.59	2.71	1.68	1.78	0.35	N
Pbk	1.95	9.51	8.09	7.91	0.25	N
Plk1	3.60	16.23	8.36	9.19	0.43	R
Pole	0.48	2.05	1.32	1.55	0.36	N
Prc1	4.36	18.71	11.24	11.77	0.39	N
Prim1	2.88	9.11	6.77	8.20	0.43	N
Racgap1	4.82	19.10	11.62	13.16	0.41	N
Rad51	1.71	5.20	3.51	3.68	0.49	N
Rad51ap1	1.52	5.91	3.61	4.27	0.42	N
Rrm2	4.84	22.29	17.55	16.90	0.29	N
Sapcd2	0.39	1.77	1.09	1.22	0.36	N
Sgol1	0.64	3.03	1.63	1.78	0.39	N
Sgol2	0.70	2.88	1.54	1.65	0.45	N
Shcbp1	1.29	6.91	4.83	4.28	0.30	N
Ska1	0.33	1.77	1.25	1.29	0.27	N
Ska3	0.83	3.67	2.02	2.22	0.41	N
Smc2	1.52	5.15	3.93	3.67	0.42	N
Snora81	0.00	1.87	1.82	1.86	0.03	N
Spag5	1.45	5.84	3.05	3.51	0.48	N
Spc24	1.83	9.19	5.56	6.14	0.33	N
Spc25	3.04	10.43	7.02	7.86	0.43	N
Spdl1	1.01	3.29	2.19	2.38	0.46	N
Tcf19	5.84	17.81	15.21	17.29	0.38	N
Tk1	3.44	19.77	14.98	17.65	0.23	N
Top2a	4.44	21.32	12.51	13.25	0.36	N
Tpx2	3.14	13.18	7.40	7.94	0.42	N
Trip13	1.35	4.02	3.00	3.03	0.45	N
Ttk	0.94	3.56	2.54	2.31	0.41	N
Uhrf1	2.56	14.19	8.68	9.82	0.29	N
Vpreb3	0.13	10.44	5.31	3.64	0.04	N
Wdhd1	0.99	3.73	2.69	2.61	0.38	N
Xpnpep2	0.71	1.63	1.74	1.60	0.44	N
1190002F15Rik	3.01	9.45	6.67	7.00	0.45	N
2700099C18Rik	1.47	5.75	3.68	3.59	0.41	M
2810417H13Rik	2.42	17.16	10.64	11.48	0.23	M
4930579G24Rik	1.88	4.55	3.92	4.04	0.48	M

FPKM, fragments per kilobase of exon per million mapped fragments
WX, irradiated *p53*$^{+/+}$ group, KX, irradiated *p53*$^{-/-}$ mice group, W, non-irradiated *p53*$^{+/+}$ group, K, non-irradiated *p53*$^{-/-}$ group.
WX/min is median WX / minimum value of median K, median KX or median W.
R, reported gene as p53 target , N, non-reported gene as p53 target, M, mouse gene which don't have human homologue.

Supplementary Table 2. Primer sequence

		Forward	Reverse
Quantitative real-time PCR	human CD137L	TCAGGCTCCGTTTCACTTG	CAGGTCCACGGTCAAAGC
	human CD137	CCTGAAGACCAAGGAGTGGA	GCAAAGCTGATTCCAAGAGAA
	human CDC42BPG	AGATGCTGAAGAGGGCTGAG	CCCCTTTCACGAGCACAT
	human FST	TCTGCCAGTTCATGGAGGA	TCCTTGCTCAGTTCGGTCTT
	mouse Cd137l	CGCCAAGCTACTGGCTAAAA	CGTACCTCAGACCTTGAGATAGGT
	mouse Cd137	TGAGCTTCTCTCCCAGTACCA	AGCAGCAAAGCCGATGTC
	mouse Cdc42bpg	GCCATTTGTTGGCTTCACTT	GGGCAGCCATTAGCTCTG
	mouse Fst	AAGCATTCTGGATCTTGCAACT	GATAGGAAAGCTGTAGTCCTGGTC
p53 binding site	human CD137L-BS-AB	AAAGGTACCTCCTTCAACACTAGCCAGTAACA	AAAAAGCTTTGGAACTACAGGCACATACCA
	mouse Cd137l-BS	AAAGGTACCTCAGTGGCTGAGAGCATTTG	AAAAAGCTTTGCTCTTAACTGCTGAACCA
	human CDC42BPG-BS-AB	AAAGGTACCCACCCAGGCTGATCTTGAAC	AAAAAGCTTTGTGTGACCTCAGGCAAGTC
	human CDC42BPG-BS-C	AAAGGTACCTGCCTGTGTTGTTGTCACC	AAAAAGCTTGGACAGGCTGCCTAATCCT
	mouse Cdc42bpg-BS	AAAGGTACCTTCTCCCTGCCTGCCTCT	AAAAAGCTTCCTGCAGAAATATCAGAGGTGA
	human FST-BS	AAACTCGAGAACAAAAATGAAAGGCGACA	AAAAGATCTGCAGCTTGGTGTTTGTTTAGTG
	mouse Fst-BS	AAAGGTACCCCTGCAGATTCATATTCATTCTC	AAAAAGCTTTTGCATTGACTTTTACTAGACTGTTT
Expression vector	human CD137L	AAAGAATTCTCTCGTCATGGAATACGCC	AAACTCGAGTTCCGACCTCGGTGAAGG
	mouse Cd137l	AAAGAATTCACCGTGGTAATGGACCAGCAC	AAACTCGAGTTCCCATGGGTTGTCGGGTTT
	human CDC42BPG	AAAGGTACCATGGAGCGGCGGCTGCGCGCG	AAAAGATCTAGGAGAGCTCTCCAATTC
	human FST317	AAAGGTACCCCCAGGATGGTCCGC	AAACTCGAGGTTGCAAGATCCGGA
	human FST344	AAAGGTACCCCCAGGATGGTCCGC	AAACTCGAGCCACTCTAGAATAGA
Reconbinant protein	human CD137	AAAGGTACCCAGGATCCTTGTAGTAACTGC	AAAGAGCTCGATCTGCGGAGAGTGTCCTGG
	mouse Cd137	AAAGGTACCCAGAACTCCTGTGATAACTGT	AAAGAGCTCAGCTGCTCCAGTGGTCTTCTT
	mouse Cd137l	AAAGAATTCACCGAGCCTCGGCCAGCGCTC	AAACTCGAGTCATTCCCATGGGTTGTCGGG
Genotyping	mouse p53	GTTATGCATCCATACAGTACA	CCGCAGGATTTACAGACACC
CHIP assay	CD137L	TCCTTCAACACTAGCCAG	TCTCAGCACTGTGATGCC
	CDC42BPG	GGCCCTCTGTTGACAATCTC	ACAGGCTGCCTAATCCTCTG
	FST	GATGCCCACAGAAAGCCTAT	TGTCTGCTCCAAATCAGCAC
siRNA		Sence	Antisense
	siP53	GACUCCAGUGGUAAUCUACTT	AGUAGAUUACCACUGGAGUCTT
	siEGFP	GCAGCACGACUUCUUCAAGT	CUUGAAGAAGUCGUGCUGC
